# Hypoxia-induced feedback of HIF-1α and lncRNA-CF129 contributes to pancreatic cancer progression through stabilization of p53 protein

**DOI:** 10.7150/thno.30988

**Published:** 2019-07-09

**Authors:** Mingliang Liu, Jianxin Zhong, Zhu Zeng, Kang Huang, Zeng Ye, Shijiang Deng, Hengyu Chen, Fengyu Xu, Qiang Li, Gang Zhao

**Affiliations:** 1 Department of Emergency Surgery, Union Hospital, Tongji Medical College, Huazhong University of Science and Technology, Wuhan 430022, China.; 2 Department of Pancreatic Surgery, Union Hospital, Tongji Medical College, Huazhong University of Science and Technology, Wuhan 430022, China.

**Keywords:** pancreatic cancer, lncRNA, HIF-1α, p53, MKRN1, FOXC2

## Abstract

**Rationale**: Emerging evidences have highlighted the critical roles of lncRNAs in human cancer development. The work sought to assess the biological role and potential underlying mechanisms of lncRNA-CF129 (CF129) which is significantly reduced in pancreatic cancer (PC).

**Methods**: CF129 expression and its association with multiple clinicopathologic characteristics in PC specimens were analyzed. The role of CF129 both *in vitro* and *in vivo* was assessed, with RNA pull-down and immunoprecipitation assays being performed to detect the interaction between CF129 and p53 and E3 ligase MKRN1. Chromatin immunoprecipitation and luciferase assays were utilized to identify the interaction between p53 and FOXC2 promoter, HIF-1α/HDAC1 complex and CF129 promoter, FOXC2 and HIF-1α promoter, respectively.

**Results**: CF129 levels were markedly lower in PC compared with paired non-tumor adjacent tissues. Low CF129 expression predicted short overall survival in PC patients. CF129 inhibited invasion and metastasis of PC cells in a FOXC2-dependent manner. In addition, CF129 regulates FOXC2 transcription through association with mutant p53. CF129 directly binds to p53 and E3 ligase MKRN1, and such an interaction leading to p53 protein ubiquitination and degradation. Furthermore, CF129 is a hypoxia-responsive lncRNA, which is transcriptionally downregulated by binding between HIF-1α/HDAC1 complex and CF129 promoter. Finally, it is revealed that HIF-1α is reciprocally regulated by FOXC2 in transcriptional level. Clinically, CF129 downregulation coordinates overexpression of FOXC2.

**Conclusions**: Our study suggests that CF129 inhibits pancreatic cell proliferation and invasion by suppression of FOXC2 transcription, which depends on MKRN1-mediated ubiquitin-dependent p53 degradation. The HIF-1α/CF129/ p53/FOXC2 axis may function as a potential biomarker and therapeutic target.

## Introduction

Pancreatic cancer is among the deadliest malignancies, with a mortality rate that ranks among the top four in worldwide. Unfortunately, there is currently no specific marker for early pancreatic cancer diagnosis, and as a result it is often diagnosed in advanced stages when there is already metastatic invasion and no opportunity for radical operation. Nevertheless, pancreatic cancer is resistance to chemotherapy and radiotherapy, which exaggerates the miserable prognosis. Because of complexities of signaling at genetic, epigenetic, and metabolic levels, the therapeutic options and progress in drug development are impeded in pancreatic cancers 1. To identify applicable biomarkers and therapeutic targets, it is extremely needed to discover novel molecular mechanisms underlying the tumorigenicity of pancreatic cancer.

Long noncoding RNAs (lncRNAs) are RNAs that are over 200 nucleotides long and do not code for protein, even though they play essential roles in many biological contexts including epigenetic regulation, cell cycle control, nuclear import, differentiation, and other pathways 2. Many lncRNAs are known to be tumor suppressors or oncogenes, further complicating the biology of human cancer 3. lncRNAs have been increasingly found to play key roles in many cancer- related processes, including cancer cell proliferation, chemoresistance, metastasis and angiogenesis 4, 5. In addition, multiple lncRNAs expression profiling are found to facilitate human cancer diagnoses 6. Therefore, lncRNAs may be able to act as either prognostic markers or targets for therapy in the context of many kinds of cancer 7. It has been reported that a series of lncRNAs are differentially expressed in pancreatic cancer and correlated with patient outcomes 8, 9. Nevertheless, the regulatory roles of dysregulated lncRNAs in PC are incompletely explored.

Many lncRNAs dysregulated in pancreatic cancer have been previously identified through microarray assay 9. Different from our previous study exploring an upregulated lncRNA, we investigated a downregulated lncRNA-CF129145.1 (CF129). We found CF129 is significantly decreased in PC tissues compared paired noncancerous peritumoral tissues. The overexpression of CF129 markedly inhibit PC cell proliferation and invasion *in vivo* and *in vitro*. Mechanical experiments further indicated that CF129 promotes an interaction of p53 and E3 ligase MKRN1, which consequently induces the ubiquitination and degradation of p53 protein in pancreatic cancers. We further revealed p53 promoted FOXC2 transcription, thereby, CF129 repressed FOXC2 expression through inducing p53 instability. Specifically, we showed CF129 was inhibited by the complex of HIF-1α and HDAC1 during hypoxia. In the meantime, FOXC2 regulates transcription of HIF-1α, by which CF129 can indirectly inhibit HIF-1α expression. Therefore, the present study demonstrates a lncRNA-interacted feedback of HIF-1α and FOXC2 which is involved in pancreatic cancer progression during hypoxia microenvironment.

## Results

### CF129 is downregulated in PC and associated with worse clinical results

**Figure 1:**
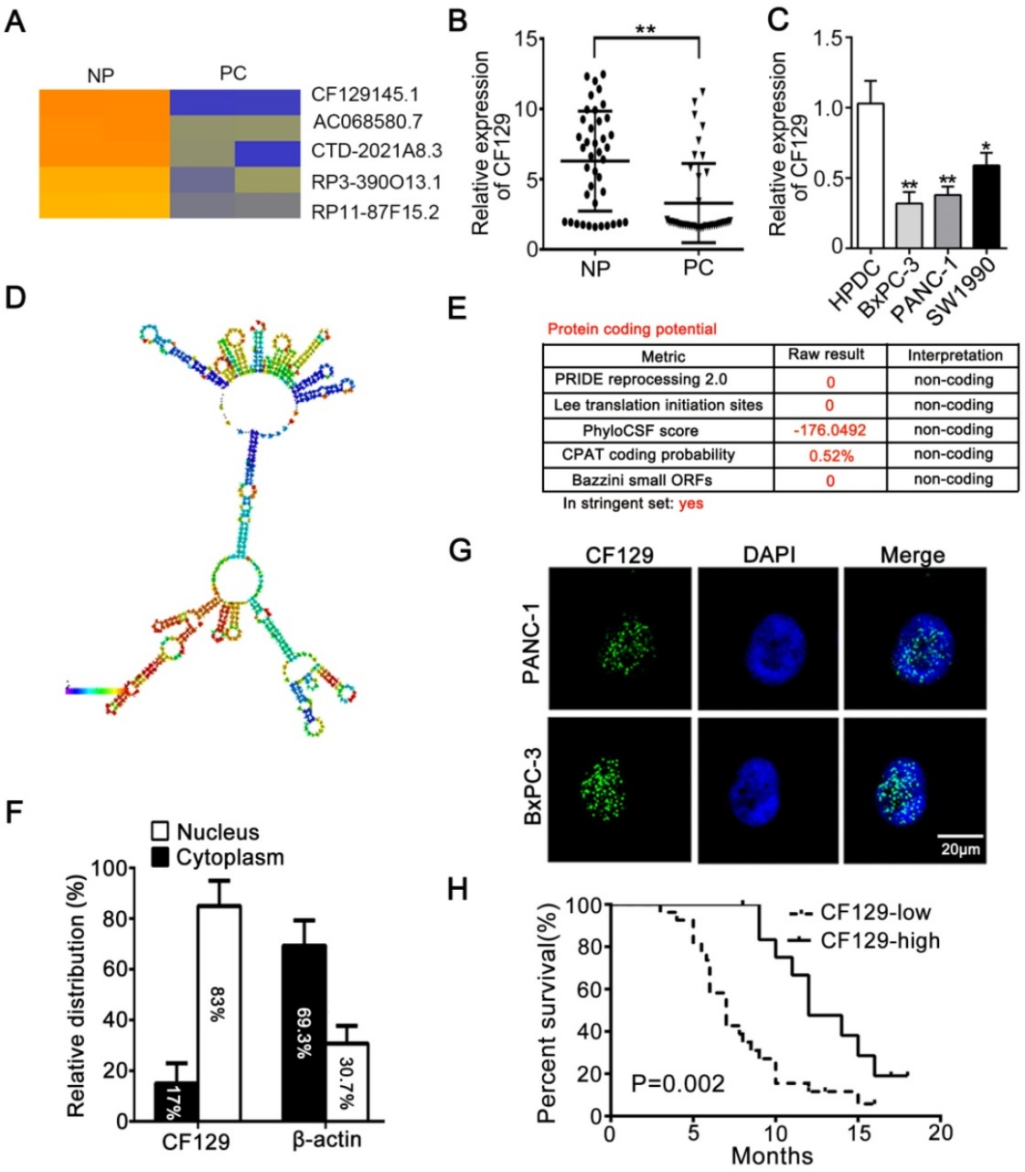
** CF129 is downregulated in pancreatic cancer and associated with worse clinical results** (A) Hierarchical clustering assessment of lncRNAs differentially expressed between normal pancreatic (NP) and pancreatic cancer (PC) tissues, which include lncRNA CF129145.1 (CF129). (B) qRT-PCR analysis of CF129 expression in 40 pairs PC and paired adjacent NP tissues. (C) CF129 expression was further compared among HPDC cells and the BxPC-3, PANC-1, and SW1990 cell lines. (D) Prediction of CF129 second structure from the database LNCipedia. Coloration indicates prediction confidence, we red corresponding to a higher degree of confidence. (E) The noncoding nature of CF129 was predicted by multiple databases from LNCipedia. (F) Total nuclear and cytoplasmic PANC-1 RNA was assessed, with relative nuclear and cytoplasmic RNA abundance shown following qRT-PCR assessment. (G) Single molecule RNA FISH detection of CF129 (green) in indicated PANC-1/BxPC-3 cells. DAPI was used to stain nuclei. Scale bars: 20μm. (H) The overall survival in 40 PC patients as assessed via Kaplan-Meier curves. The cut-off for determination of high/low CF129 expression was the median overall expression level. Groups were compared via Log-rank test.

Differential lncRNA expression between pancreatic cancer (PC) and paired noncancerous peritumoral (NP) tissues determined via microarray, revealing lncRNA-CF129 (CF129) to be among the most significantly decreased lncRNAs in PC samples (**Figure 1A**). Moreover, the downregulation of CF129 was further verified in 40 cases of PC tissues relative to paired NP tissues (**Figure 1B**). Compared to immortalized human pancreatic duct cells (HPDC), CF129 was downregulated in most of the cell lines and PANC-1 and BxPC-3 cells were the lowest (**Figure 1C**). Meanwhile, the secondary structure of CF129 is predicted by online database AnnoLnc 10 (**Figure 1D**). In addition, the protein-coding capacity of CF129 was evaluated by online prediction software, which validated CF129 as a non-coding RNA (**Figure 1E**). Furthermore, results from nuclear/cytoplasmic RNA fractionation revealed that CF129 mainly presents in the nucleus (**Figure 1F**). This result was further confirmed via fluorescence in situ hybridization (FISH) using a CF129 probe (**Figure 1G**). We next evaluated the relationship between CF129 expression and the clinicopathologic characteristics of PC patients. The correlation analysis showed that low expression of CF129 was associated with large tumor size, poor tumor differentiation, lymphatic invasion, distant metastasis (**Supplementary Table 1**), and shorter overall survival (**Figure 1H**). This suggested CF129 might be involved in PC development.

### CF129 overexpression inhibits PC cell proliferation and invasion

**Figure 2:**
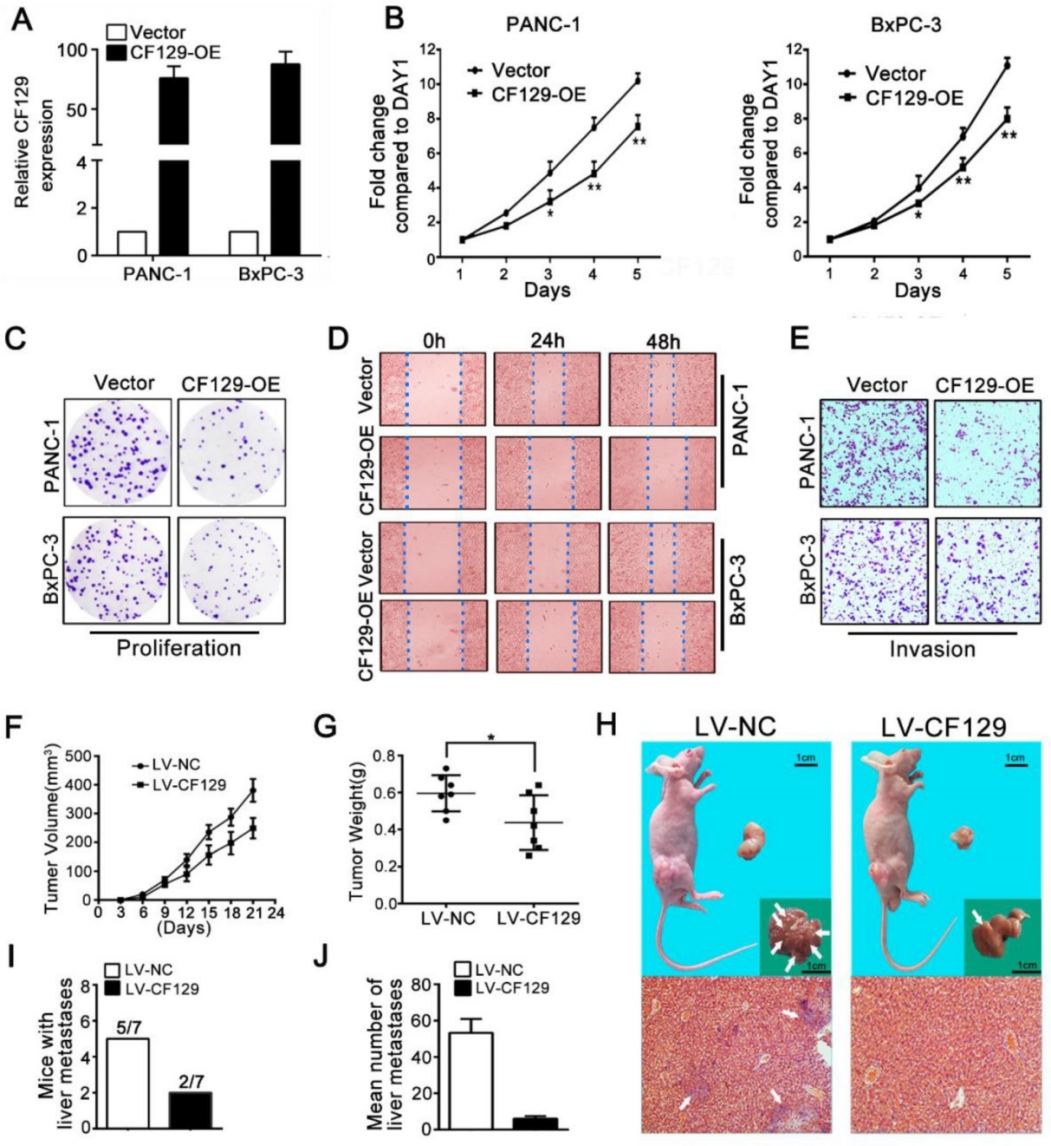
** CF129 overexpression inhibits PC cell proliferation and invasion.** (A) CF129 expression was detected in PANC-1 and BxPC-3 cells by qRT-PCR following transfection with a control plasmid or one encoding full-length human CF129. (B, C) Proliferation of PANC-1/BxPC-3 cells overexpressing CF129 or control were assessed via MTT assays for 5 days and colony formation assay for 8 days. (D, E) How CF129 overexpression affects cellular migration and invasion ability was investigated by using wound-healing and transwell assays. (F) PANC-1 cells transfected with control and CF129 overexpression vector were implanted in the flanks of nude mice and tumor volumes were assessed every third day (n=7 mice per group). (G) After 3 weeks, the weight of each tumor was measured respectively. (H) Tumor, liver, and H&E stained sections are shown. Arrows indicate the invasion nodules. (I, J) The frequency and number of liver metastases in mice were quantified. Overexpressed CF129 results in lower incidence of liver metastases (5/7 vs 2/7) and fewer total metastases (53 vs 5).

Since CF129 was downregulated in PC tissues, we explored its biology by overexpression of CF129. After transfection with CF129-containing vector (CF129-OE) (**Figure 2A**), the PANC-1/BxPC-3 proliferation and colony formation were remarkably suppressed (**Figure 2B-C**). Additionally, both the wound-healing and transwell assays demonstrated suppression of migration and invasion of PC cells due to CF129 overexpression (**Figure 2D-E**).

To validate these findings, PANC-1 cells were transfected stably using lentivirus containing negative control (LV-NC) or CF129 sequences (LV-CF129) and further implanted into nude mice to construct xenograft tumor model. Relative to LV-NC mice, the tumors in LV-CF129 mice were smaller and weighed less (**Figure 2F-G**). In addition, the number of mice with liver metastases and the visible metastasis loci in the LV-CF129 group was apparently less than those in the control group (**Figure 2H-J**).

To further elaborate impacts of CF129 on pancreatic cancer cells, three siRNAs targeting CF129 were designed and siCF129-1 and siCF129-2 were used for experiments (**Figure S1A**). Contrary to CF129 overexpression, downregulation of CF129 remarkedly promoted proliferation, colony formation of PANC-1/BxPC-3 cells (**Figure S1B-C**). In addition, migration and invasion were also significantly increased following transfection with siCF129 (**Figure S1D-E**).

### CF129 inhibits FOXC2 expression in pancreatic cancer cells

**Figure 3:**
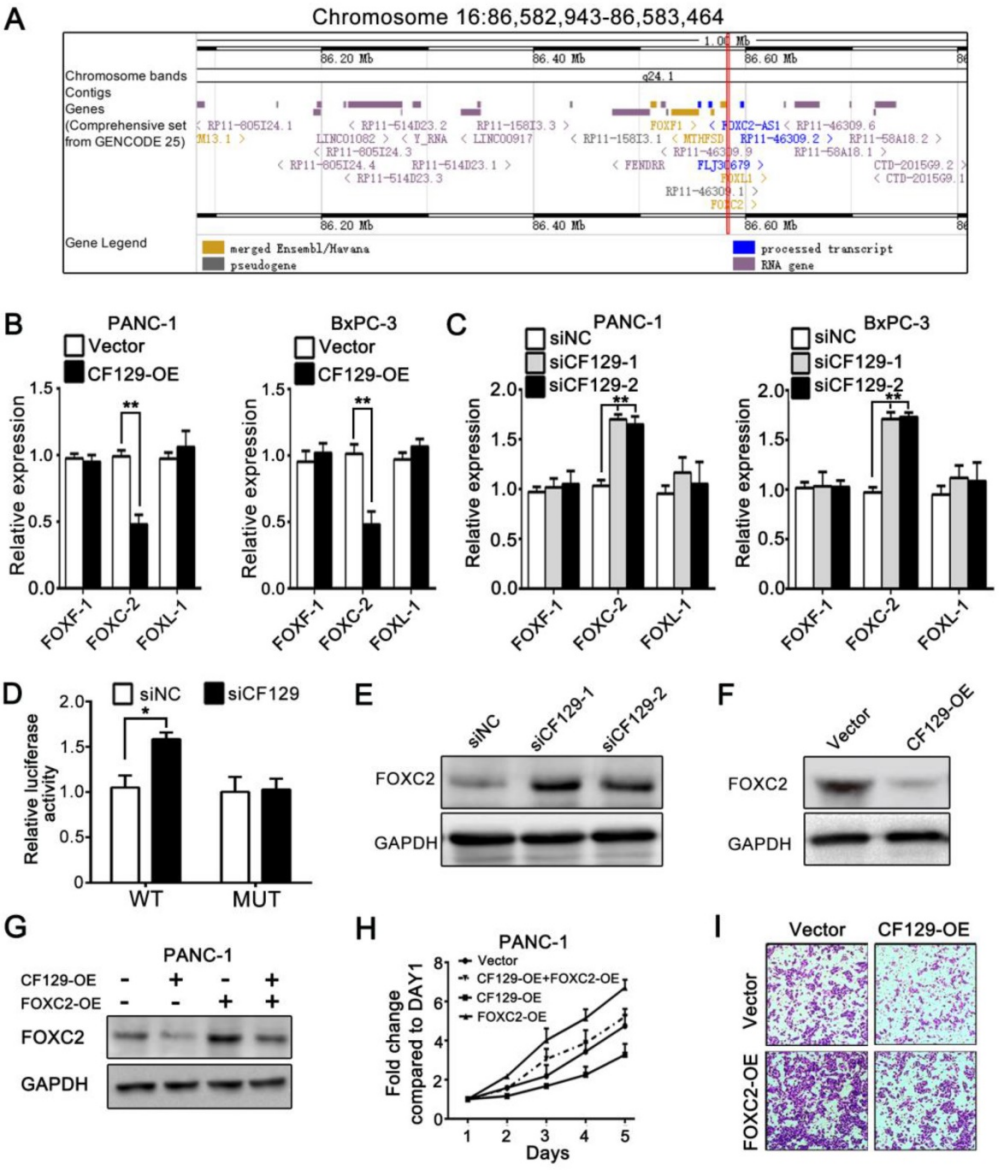
** CF129 inhibits FOXC2 expression in pancreatic cancer cells.** (A) Schematic annotation of genomic locus of CF129 and its adjacent gene including FOXC2, FOXF1 and FOXL-1. (B) qRT-PCR was used to measure mRNA levels of FOXC2, FOXF1 and FOXL-1 in CF129-overexpressing PANC-1/BxPC-3 cells. (C) The mRNA levels of FOXC2, FOXF1 and FOXL-1were detected in CF129-knockdown PANC-1 and BxPC-3 cells by qRT-PCR. (D) After PANC-1 cells underwent transfection using WT or MUT plasmids, they were co-transfected using siCF129 or siNC, respectively. The luciferase density was measured and plotted after normalizing. Shown data are mean±SD activity. (E, F) FOXC2 protein was detected in CF129-knockdown or -overexpression PANC-1 cells via western blotting. (G) FOXC2 protein was measured in PANC-1 cells transfected using CF129-OE or/and FOXC2-OE by western blot analysis. (H) Proliferation of PANC-1 cells transfected using CF129-OE or/and FOXC2-OE were compared by MTT assay. (I) Transwell assay displayed the invasion ability of PANC-1 cells transfected using CF129-OE or/and FOXC2-OE, respectively.

Researches had demonstrated that lncRNAs regulate expression of neighborhood gene in cis. Since CF129 is adjacent to forkhead family of transcription factors including FOXF1, FOXC2 and FOXL1 (**Figure 3A**), we further explore whether CF129 could regulate expression of FOXF1, FOXC2 or FOXL1. Interestingly, CF129 overexpression or knockdown dramatically inhibited or increased expression of FOXC2 mRNA, but not expression of FOXF1 and FOXL1 mRNA (**Figure 3B- C**). Moreover, the luciferase reporter assay showed that siCF129 obviously increased the luciferase intensity of PANC-1 cells which were transfected with vector containing FOXC2 promoter (**Figure 3D**). Coincidently, expression level of FOXC2 protein was significantly increased by siCF129 but decreased with CF129 overexpression (**Figure 3E-F**). As that of CF129 overexpression, FOXC2 knockdown with siFOXC2 dramatically suppressed proliferation and invasion of PANC-1 and BxPC-3 cells (**Figure S2A-C**). Furthermore, the co-transfection with FOXC2-OE effectively rescued FOXC2 expression, proliferation, and invasion of PANC-1 cells which were inhibited by CF129 overexpression (**Figure 3G-I**). On the contrary, siFOXC2 dramatically depleted FOXC2 expression, proliferation and invasion of PANC-1 cells which were induced by siCF129 (**Figure S2D-F**). These results intensively suggest that CF129 exerts function in pancreatic cancer cells by repressing FOXC2 expression in transcriptional level.

### CF129 regulates FOXC2 transcription through association with p53

**Figure 4:**
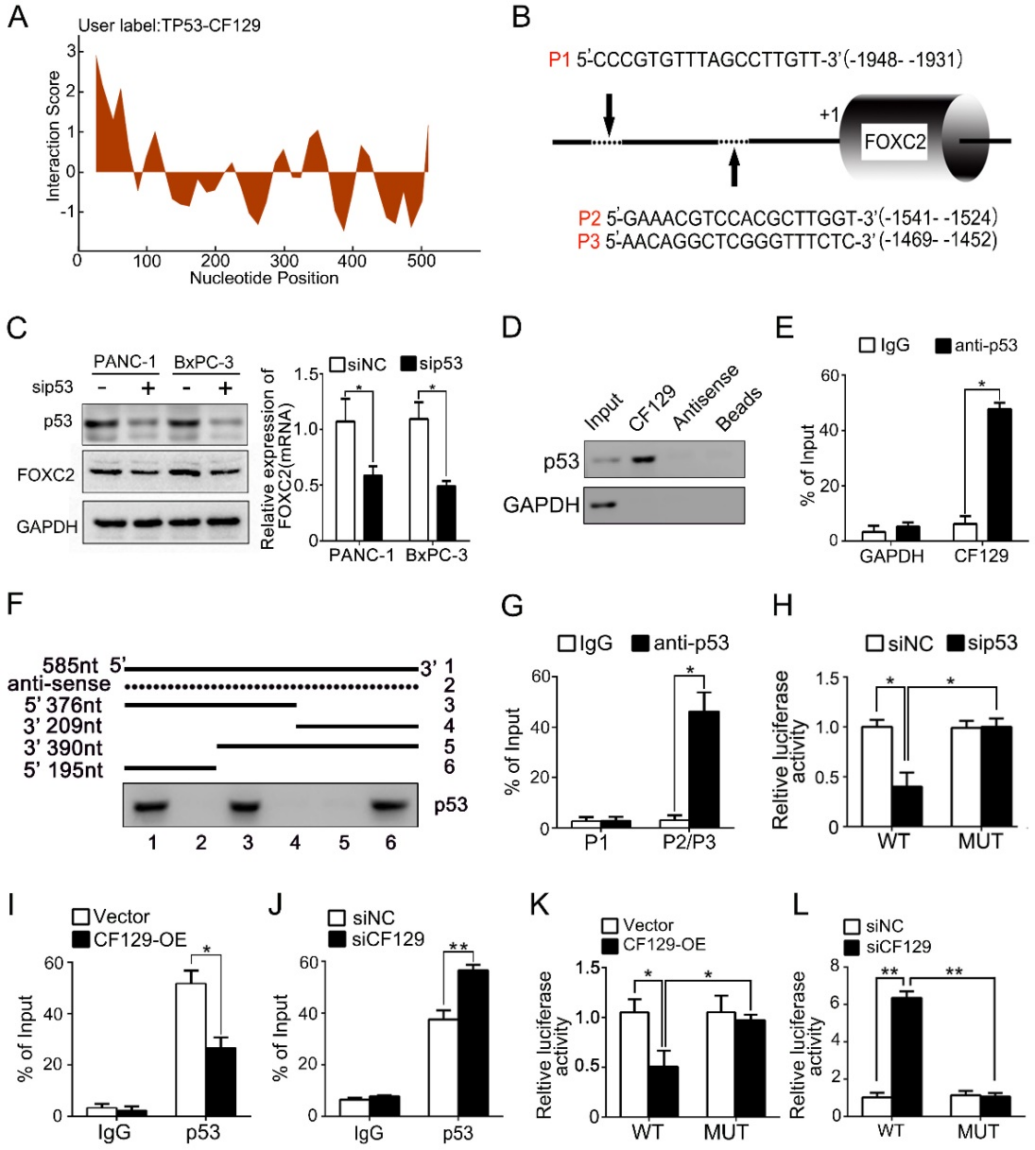
** CF129 regulates FOXC2 transcription through association with p53.** (A) Schematic illustration of binding sites between CF129 and p53 protein. Coloration indicates prediction confidence, we red corresponding to a higher degree of confidence. (B) Schematic illustration showed p53 responsive elements (P1, P2 and P3) on the FOXC2 promoter. (C) p53 and FOXC2 mRNA/protein were assessed in p53-knockdown PANC-1/BxPC-3 cells via qRT-PCR and western blotting. (D) Lysates from PANC-1 cells were used for RNA-pulldown with biotinylated CF129 transcript or antisense transcript. Western blot analysis of the specific association of p53 and CF129 with ant-p53 antibody. GAPDH was used as a control. (E) PANC-1 lysates underwent RIP using anti-p53 or anti-IgG, and CF129 was measured via qRT-PCR. GAPDH mRNA was used as negative controls. (F) PANC-1 lysates were mixed with biotinylated CF129 RNA or antisense (dotted line) fragments, and bound proteins were assessed via western blotting to measure p53 binding to CF129. (G) Lysates of PANC-1 cells were used for ChIP with anti-p53, with resultant products being amplified via PCR. (H) PANC-1 cells were transfected with WT or MUT reporter plasmid, and further co-transfected with sip53 respectively. The luciferase density was measured and plotted after normalizing. (I, J) After transfection using CF129-OE or siCF129, PANC-1 cells were used for ChIP analyses. (K, L) The reporter PANC-1 cells were co-transfected using CF129-OE or siCF129, and the luciferase density were measured and normalized. Shown data are mean±SD (n = 3).

lncRNAs have recently been found to exert effects in part via interacting with transcription factors. To predict the putative transcription factor involved in regulation of CF129 on FOXC2, an intersection analysis of online database catRAPID and Jaspar was applied. The catRAPID analysis showed high affinity between CF129 transcript and p53 protein (**Figure 4A**). Moreover, the results of Jaspar revealed 3 putative binding sits for p53 in the FOXC2 promoter region (**Figure 4B**). Meanwhile, knockdown of p53 dramatically inhibited FOXC2 expression both in mRNA and protein level (**Figure 4C**). Therefore, we assumed that p53 may act as transcription factor to mediate the regulation of CF129 on FOXC2 expression. To further identify the association of CF129 and p53, we performed RNA pulldown and RNA immunoprecipitation (RIP) assays. The western blot results showed that p53 protein was accumulated by CF129 but not by antisense transcript (**Figure 4D**). The qPCR results confirmed that CF129 was significantly enriched by anti-p53 antibody but not IgG (**Figure 4E**). In addition, deletion-mapping analyses identified that the 195nt region at the 5' end of CF129 is required for its association with p53 (**Figure 4F**). In addition, the ChIP assay verified the association of p53 and the chromatin fragments corresponding to the site P2/P3, rather than the site P1 within the FOXC2 promoter region (**Figure 4G**). Furthermore, we used luciferase reporter assays to validate whether p53 activating transcription of FOXC2 promoter. FOXC2 promoter sequence containing wild-type (WT) or mutant-type P2/P3 (MUT) was inserted in the luciferase reporter plasmid promoter. We found that luciferase activity from the WT, but not the MUT, was markedly decreased by p53 knockdown (**Figure 4H**).

Moreover, the CF129 overexpression markedly decreased, while the CF129 knockdown increased the enrichment of p53 on FOXC2 promoter (**Figure 4I-J**). Coincidently, the luciferase activity of WT was decreased by CF129 overexpression, but increased by CF129 downregulation (**Figure 4K-L**). Coincidently, the siCF129- induced upregulation of FOXC2 was inhibited by the knockdown of p53 in BxPC-3 cells (**Figure S3A-B**). Importantly, the enhanced proliferation and invasion abilities of CF129-knockdown BxPC-3 cells were reversed by co-transfection with sip53 (**Figure S3C-D**). Since both BxPC-3 and PANC-1 cell lines are p53 mutant phenotype 11, our results suggested that the mutant p53 might be required for the transcriptional regulation of CF129 on FOXC2.

### CF129 induces ubiquitination and degradation of p53

**Figure 5:**
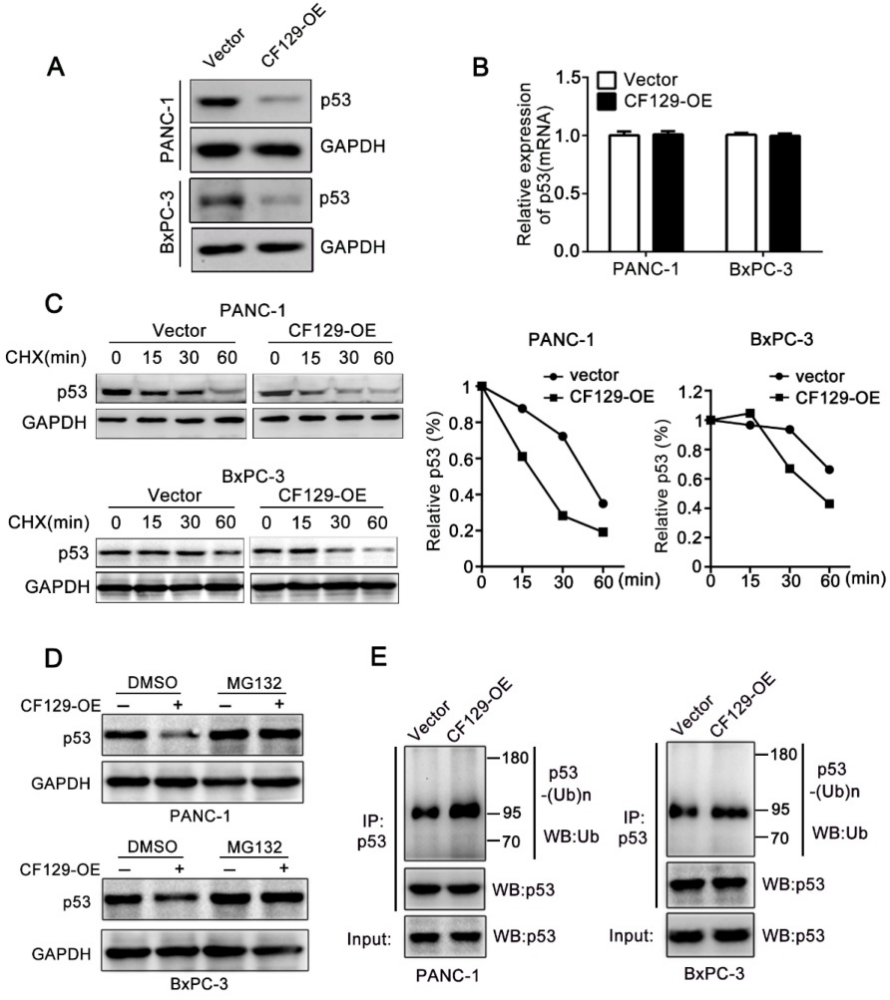
** CF129 induces p53 ubiquitination and degradation.** (A) p53 protein levels were assessed in PANC-1/BxPC-3 cells transfected using CF129-OE by Western blot analysis. (B) qRT-PCR assessment of p53 levels in PANC-1/BxPC-3 cells transfected with CF129-OE. (C) After transfected with CF129-OE, CHX (100 ug/mL) was used to treat PANC-1/BxPC-3 cells prior to western blotting, with ImageJ used to quantify p53 band densitometry. (D) After transfected with CF129-OE, PANC-1 and BxPC-3 cells were treated with MG132(20nM) for 3h and used for western blotting. (E) The CF129-OE transfected PANC-1 cells were treated using MG132(20nM) for 3h. Anti-p53 was then used for immunoprecipitation of these cell lysates, followed by western blotting to assess endogenous p53 ubiquitination.

Researches had reported that lncRNA regulate protein degradation and thereby plays a critical role in tumorigenicity 12, 13. Therefore, we further investigated whether CF129 could regulate the stability of p53 protein. Interestingly, we revealed that CF129 overexpression significantly attenuated (**Figure 5A**), while CF129 knockdown enforced expression of p53 protein (**Figure S4A**). Nevertheless, neither siCF129 nor CF129-OE demonstrated obvious effects on mRNA expression of p53 (**Figure 5B and Figure S4B**). To further reveal whether CF129 regulated p53 stability, p53 protein was measured in cycloheximide (CHX) treated cells, to prevent protein synthesis. The results showed that p53 protein in pancreatic cancer cells was destabilized with CF129 overexpression (**Figure 5C**), while knockdown of CF129 increased the stability of p53 protein (**Figure S4C**).

Moreover, the proteasome inhibitor MG132 could abolish the regulation of CF129 overexpression (**Figure 5D**) or downregulation on p53 expression (**Figure S4D**). Given that p53 is regulated by the ubiquitin-proteasome pathway 14, we further explored whether the CF129-induced degradation was ascribed to ubiquitination. Coincidently, we found that overexpression of CF129 increased ubiquitination of p53 (**Figure 5E**), while knockdown of CF129 decreased ubiquitination of p53 (**Figure S4E**), respectively. Together, these data suggest that CF129 inhibits p53 protein expression in post- transcriptional level by promoting ubiquitin- dependent degradation.

### MKRN1 is required for the CF129-mediated p53 degradation

**Figure 6:**
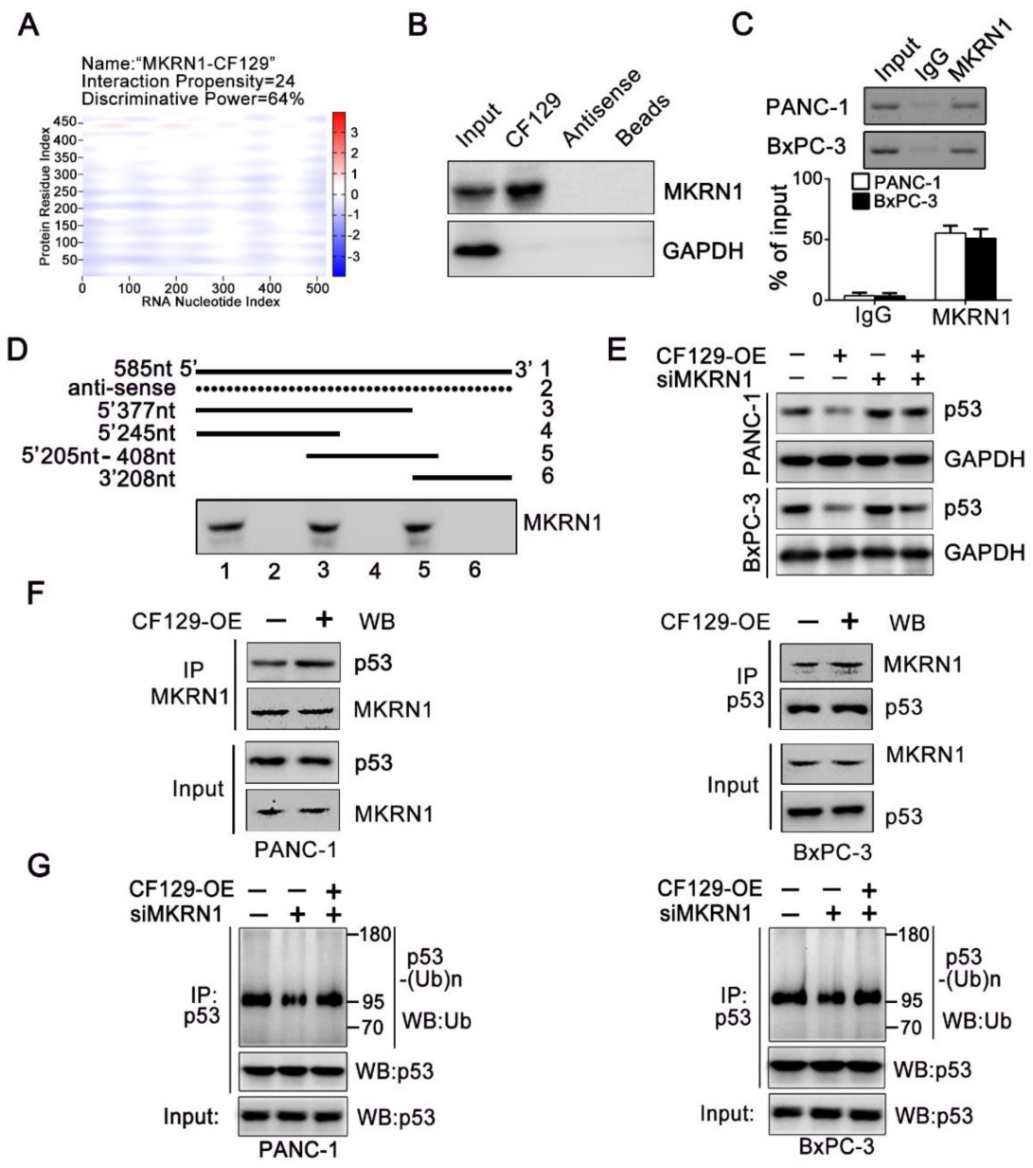
** MKRN1 is required for the CF129-mediated p53 degradation.** (A) Schematic illustration of binding sites between CF129 and MKRN1. The segment, which has the highest peak, is most likely to bind to MKRN1. (B) The RNA-pulldown was applied with cell extracts of PANC-1 cells by using biotinylated CF129 transcript or antisense RNA. The specific association of MKRN1 and CF129 were assessed via western blotting using anti-MKRN1 antibody. (C) Lysates from PANC-1 and BxPC-3 cells underwent immunoprecipitation using anti-MKRN1 antibody or control IgG and CF129 levels were measured via qRT-PCR. (D) Biotinylated CF129 or antisense (dotted line) fragments were combined with PANC-1 whole cell extracts and performed for RNA-pulldown. The associated MKRN1 protein was assessed by western blotting. (E) Western blotting of p53 expression in PANC-1/BxPC-3 cells after CF129-OE or/and siMKRN1 transfection. (F) After CF129-OE transfection, the lysates from PANC-1/BxPC-3 cells were treated using MG132, and immunoprecipitated using anti-MKRN1 or anti-p53 followed by western blotting with anti-MKRN1 or anti-p53. (G) After siMKRN1 or/and CF129-OE transfection, the lysates from PANC-1/BxPC-3 cells were MG132 treated and used for anti-p53 immunoprecipitation followed by western blotting with an anti-ubiquitination antibody.

We further explore the precise mechanism about how CF129 regulates the ubiquitin-dependent degradation of p53 protein. Interestingly, depending on the online prediction from catRAPID, Makorin Ring Finger Protein 1 (MKRN1), a E3 ligase, might be associated with CF129 (**Figure 6A**). It has been reported that MKRN1 induces the ubiquitination and proteasomal degradation of p53 15. Meanwhile, the Co-IP analyses validated an association between MKRN1 and p53 in both PANC-1 and BxPC-3 cells (**Figure S5A**). A significant upregulation of p53 protein upon MKRN1 knockdown was detected in both PANC-1 and BxPC-3 cells (**Figure S5B**). Based on the findings, we presumed that MKRN1 might be involved in the process about CF129- induced degradation of p53. RNA pulldown assays using lysates from PANC-1 cells confirmed the association between CF129 and MKRN1 (**Figure 6B**). The association of CF129 and MKRN1 was further confirmed by RIP analysis with anti-MKRN1 antibody (**Figure 6C**). Deletion-mapping analyses identified that the segment (5' 246-377nt 3') of CF129 were required for the association with MKRN1 (**Figure 6D**). Coincidently, MKRN1 knockdown abrogated the decrease of p53 protein caused by the CF129 overexpression in PANC-1 and BxPC-3 cells (**Figure 6E**). Vice versa, knockdown of CF129-induced p53 expression was rescued by overexpression of MKRN1 (**Figure S5C**). We then further evaluated whether CF129 modulated the association between MKRN1 and p53. Co-IP assays revealed that overexpression of CF129 promoted the interactions between MKRN1 and p53 (**Figure 6F**). On the contrary, the interaction between MKRN1 and p53 was reduced upon the CF129 knockdown (**Figure S5D**). In addition, MKRN1 knockdown dramatically decreased p53 ubiquitination, which was increased after co-transfected with CF129 (**Figure 6G**). In contrast, MKRN1-enhanced ubiquitination of p53 was reduced by CF129 knockdown (**Figure S5E**). Interestingly, the knockdown of MKRN1 did not affect the interaction between CF129 and p53, whereas the knockdown of p53 also did not affect the association between CF129 and MKRN1 (**Figure S5F**).

Together these results indicate that CF129 promotes the association of MKRN1 and p53, thereafter enhancing MKRN1-mediated p53 ubiquitination and subsequent degradation.

### CF129 is downregulated by HIF-1α during Hypoxia

**Figure 7:**
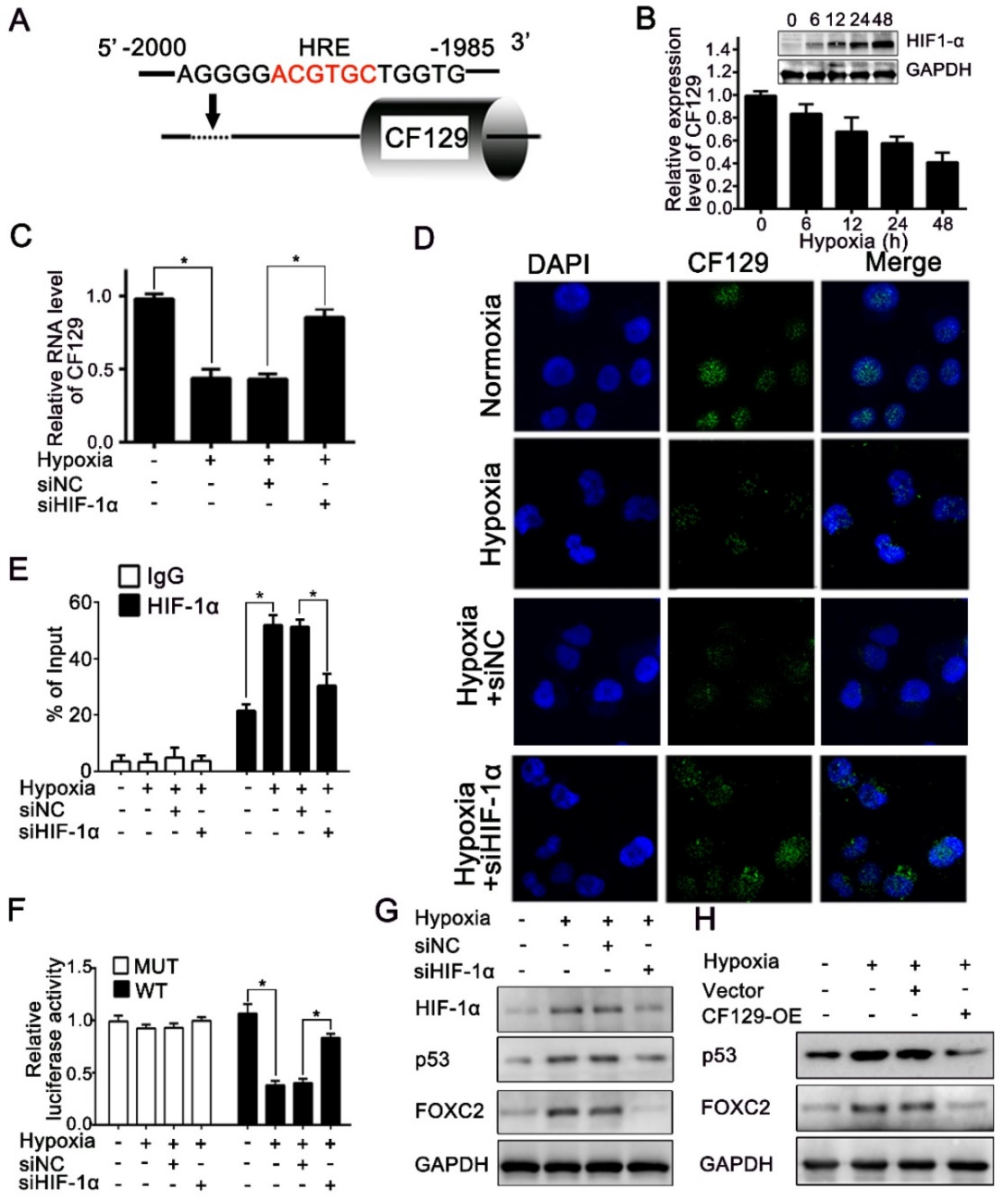
** CF129 is downregulated by HIF-1α during Hypoxia.** (A) A putative HREs (ACGTGC) were found in the promoter of CF129 gene. (B) PANC-1 cells were cultured during hypoxia, and CF129 mRNA and HIF-1α protein were measured via qRT-PCR and western blotting, respectively, at indicated timepoints. (C) CF129 expression of siHIF-1α-transfected PANC-1 cells was compared to hypoxic siNC-transfected PANC-1 cells via qRT-PCR. (D) Single molecule RNA FISH detection of CF129 (green) in hypoxic or normoxic PANC-1 cells with siNC or siHIF-1α transfection. Nucleus was counterstained with DAPI. (E) ChIP was used to assess hypoxic or normoxic PANC-1 cells transfected with siNC or siHIF-1α by using anti-HIF-1α antibody. (F) PANC-1 cells underwent transfection using WT or MUT plasmid as reporter cells, which were further transfected using siNC or siHIF-1α under normoxia or hypoxia. The luciferase activity was measured and normalized. (G) Western blotting detection of HIF-1α, p53, and FOXC2 in hypoxic or normoxic PANC-1 cells transfected with siNC or siHIF-1α. (H) The protein level of PANC-1 cells transfected with control or CF129-OE under normoxia or hypoxia was analyzed by Western blot.

Hypoxia is a common feature of solid tumors and plays key roles in tumor development. Recent results had shown lncRNAs were aberrantly expressed during hypoxia 16. Given the promoter area of CF129 gene containing a potential hypoxia responsive element (HRE) sequence (-2000 to -1985nt) (**Figure 7A**), we next explored whether CF129 was transcriptionally regulated by HIF-1α during hypoxia. Coincidently, CF129 were significantly downregulated with treatment of hypoxia (**Figure 7B**) or COCl2 (**Figure S6A**) in a time-dependent manner. Moreover, both qRT-PCR and FISH assays demonstrated that HIF-1α knockdown obviously increased CF129 expression which was repressed by the hypoxia (**Figure 7C-D**) or COCl2 treatment (**Figure S6B-C**). Coincidently, the ChIP assays validated an association between CF129 promoter and HIF-1α was enhanced by hypoxia or COCl2 but suppressed after transfection with siHIF-1α (**Figure 7E and Figure S6D**). Additionally, luciferase reporter plasmid containing CF129 promoter (wild type, WT) or HRE-mutant CF129 promoter (mutant type, MUT) was transfected into PANC-1 and BxPC-3 cells. The results verified the siHIF-1α enhanced luciferase intensity which was inhibited by hypoxia (**Figure 7F**) or COCl2 treatment (**Figure S6E**).

The expression of p53 and FOXC2 was further evaluated to validate the critical roles of CF129 during hypoxia. The western blot results showed that both siHIF-1α and CF129 overexpression could reverse p53 and FOXC2 expression which was induced by hypoxia (**Figure 7G-H**) or COCl2 (**Figure S6F-G**). Since CF129 is a hypoxia-responsive lncRNA, we further identified the effects of CF129 on growth of PC cells during different oxygen concentration. Similar to 5% O2 (**Figure S7A**), the growth of PANC-1 cells were significantly promoted by CF129 depletion but inhibited by CF129 overexpression during both 1% O2 and 0.5% O2 (**Figure S7B-C**). Therefore, these data intensively indicate CF129 is a hypoxia-responsive lncRNA in pancreatic cancer.

### Histone deacetylase 1 (HDAC1) is involved in the HIF-1α-induced reduction of CF129 expression

Research has revealed that HDACs are involved in downregulation of noncoding RNA including lncRNA and microRNA under hypoxic condition 17, 18. In present study, we investigated whether HDAC1 is required for the repression of CF129 under hypoxia. The siHDAC1-1 and -2 with effective inhibition on HDAC1 expression were applied for the further experiments (**Figure S8A**). PANC-1/BxPC-3 CF129 expression during hypoxia was obviously upregulated by treatment of HDAC1 knockdown or deacetylase inhibitor trichostatin A (TSA) (**Figure S8B-C**). Meantime, the ChIP assay displayed the hypoxia-enhanced binding of HDAC1 with HRE of CF129 promoter was significantly downregulated by siHDAC1 but not TSA (**Figure S8D-E**). Nevertheless, the hypoxia-inhibited luciferase density of WT reporter was upregulated by siHDAC1 or TSA (**Figure S8F-G**). Coincidently, we also revealed that hypoxia-induced p53 and FOXC2 was markedly repressed by siHDAC1 or TSA (**Figure S8H-I**). Therefore, these results indicate that the downregulation of CF129 is regulated via the HIF-1α/HDAC1 complex in hypoxic contexts.

### HIF-1α is reciprocally regulated by FOXC2 in transcriptional level

**Figure 8:**
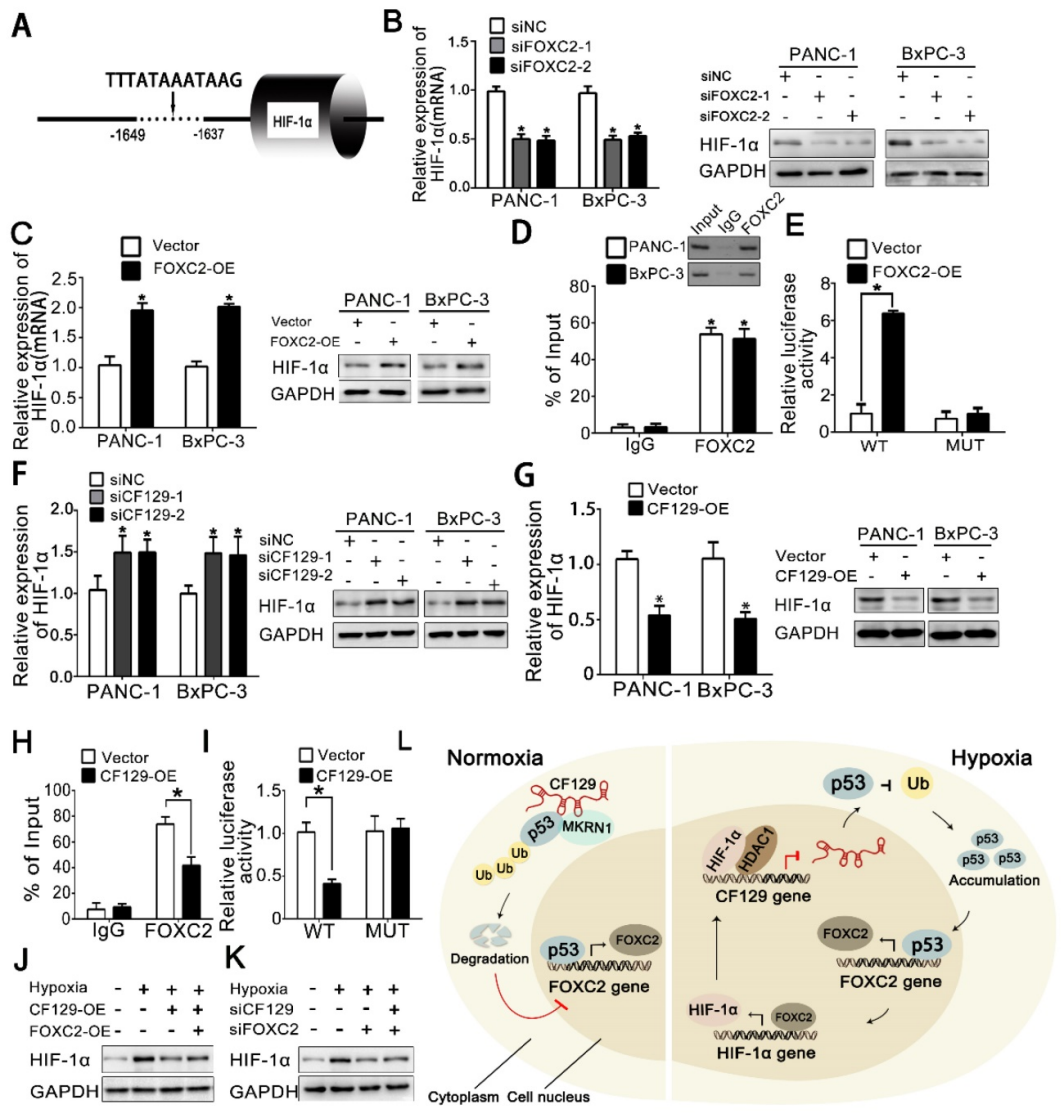
** HIF-1α is reciprocally regulated by FOXC2 in transcriptional level** (A) Schematic illustration of FOXC2 binding site on the HIF-1α promoter region. (B, C) RNA and protein levels of HIF-1α were measured in hypoxic PANC-1/BxPC-3 cells treated with siFOXC2 or FOXC2-OE. (D) ChIP assessments of PANC-1/BxPC-3 cells with anti-FOXC2 antibody. qRT-PCR of ChIP assay showing the accumulation of HIF-1α promoter bound to the FOXC2. (E) PANC-1 cells underwent transfection using MUT or WT plasmid as reporter cells. The luciferase activity was measured and normalized. (F, G) HIF-1α levels were detected hypoxic PANC-1/BxPC-3 cells following siCF129 and CF129-OE transfection. (H) ChIP assessment of PANC-1 cells transfected using vector or plasmid targeting CF129 via anti-FOXC2 antibody. (I) PANC-1 cells transfected using WT or MUT plasmid as reporter cells, were transfected with vector or CF129-OE. Luciferase activity was measured and normalized. (J) Protein levels of HIF-1α were assessed in PANC-1 cells treated using CF129-OE or/and FOXC2-OE during hypoxia. (K) Protein levels of HIF-1α were detected in PANC-1 cells treated with siFOXC2 or/and siCF129 during hypoxia. (L) The graphical representation of the reciprocal feedback of CF129-mediated HIF-1α and FOXC2 in pancreatic cancer. * P< 0.05,**P<0.01.

Based on that FOXC2 is a transcriptional factor, DNA sequence analysis showed a putative HRE (-1649 to -1639nt) in the HIF-1α promoter region (**Figure 8A**), which implied HIF-1α might be regulated by FOXC2 in transcriptional level. As expected, both HIF-1α mRNA and protein expression was significantly reduced after knockdown of FOXC2 (**Figure 8B**) but increased by overexpression of FOXC2 (**Figure 8C**). Coincidently, the ChIP assay demonstrated FOXC2 antibody induced obvious accumulation of HIF-1α promoter (**Figure 8D**). To further validate whether FOXC2 binding on the HIF-1α promoter was functional, HIF-1α promoter containing wild-type (WT) or mutant (MUT) FOXC2-binding sequence was inserted into the luciferase reporter plasmid. Results showed the luciferase density of the WT reporter, but not the MUT reporter, was markedly induced by FOXC2 overexpression (**Figure 8E**).

Since FOXC2 is repressed by CF129, we further presumed whether HIF-1α can be reciprocally regulated by CF129. Results showed CF129 knockdown increased, while CF129 overexpression decreased both mRNA and protein level of HIF-1α in PANC-1/BxPC-3 cells (**Figure 8F-G**). Moreover, the ChIP and luciferase reporter assay demonstrated that CF129 overexpression significantly inhibited the accumulation and activation of CF129 on FOXC2 promoter (**Figure 8H-I**). Meantime, the hypoxia- induced HIF-1α was dramatically attenuated by CF129 but rescued by the FOXC2 overexpression (**Figure 8J**). Vice versa, the siCF129 markedly compensated the HIF-1α expression which was inhibited by siFOXC2 during hypoxia (**Figure 8K**).

Taken together, the above data suggests that CF129/FOXC2 pathway can reciprocally regulation of HIF-1α expression during hypoxia, thereby, forming a feedback loop.

### Clinical correlation between CF129 and FOXC2 in PC

To further assess the regulation of CF129 on FOXC2 expression, we measured FOXC2 mRNA level in the same cohort of the 40 pancreatic cancer tissues. The FOXC2 level was significantly higher in PC tissues than that in (NP) tissues (**Figure S9A-B**). Moreover, the publicly available databases for pancreatic cancer (GEO profiles, GDS4102) also displayed that FOXC2 was significantly higher in human PC tissues than that of NP tissues (**Figure S9C-D**). Furthermore, a significant inverse correlation was observed between CF129 and FOXC2 transcript levels in 40 pancreatic cancer specimens (r=-0.723, p<0.039) (**Figure S9E**). Moreover, the online database (ChIPbase) demonstrated an obvious correlation between HIF-1α and FOXC2 mRNA expression in pancreatic adenocarcinoma (PDDA) (**Figure S9F**). Kaplan-Meier and log-rank test analyses showed that pancreatic cancer patients with high FOXC2 expression had a shorter overall survival time than those patients with low FOXC2 expression (**Figure S9G**). Based on our results, we depicted a schematic diagram to depict that CF129 played an important role in regulation of HIF-1α/FOXC2 pathway during hypoxia stress (**Figure 8L**).

Therefore, these data further indicated that the dysregulated CF129/FOXC2 pathway is involved in the development of pancreatic cancer.

## Discussion

While thousands of lncRNAs are known, the functional roles of the majority of these molecules are unknown, particularly in the context of pancreatic cancer. We employed a microarray for lncRNA that revealed a dysregulated lncRNA-CF129, the downregulation of which was further validated in pancreatic cancer patients. We further verified CF129 function as an inhibitor by repressing FOXC2 transcription. Mechanically, our present study showed that CF129 mediated an association of p53 and MKRN1, an E3 ligase, which inducing p53 ubiquitination and subsequent degradation. Consequently, CF129 inhibits the FOXC2 transcriptional activity on p53, by which retard the proliferation and metastasis of PC cells. Specifically, we demonstrated a negative loop between CF129 and HIF-1α: HIF-1α/HDAC1 transcript inhibits CF129 transcription, while CF129-induced repression of FOXC2 reciprocally represses HIF-1α transcription by regulating FOXC2 expression. Agree with the experimental results, low CF129 expression in pancreatic cancer was significantly associated with numerous clinicopathologic characteristics, including larger tumor size, poor differentiation, lymphatic invasion, and distant metastasis. All these data support our conclusion that CF129 is an inhibitor on development of pancreatic cancer, thus highlighting it as a potential target for PC treatment.

lncRNAs found in the nuclear compartment of cells are often involved in the regulation of chromatin, transcriptional activity, or RNA processing, exerting gene-specific effects at the chromatin level both in cis and in trans. Since CF129 is adjacent to transcriptional factor forkhead proteins family (FOX) including FOXF1, FOXC2 and FOXL1, we further investigated whether CF129 exerts its function by regulating these FOX proteins expression in cis. Interestingly, knockdown or overexpression of CF129 obviously decreased or increased FOXC2 expression, without effects on FOXF1 and FOXL1 expression. FOXC2 has been reported to be overexpressed in numerous cancers and function as a central regulator for epithelia-mesenchymal transition (EMT) and metastasis, including breast cancer, lung cancer, colorectal cancer, et al^19^-^22^. Thus, we presumed that FOXC2 might be a relevant CF129 target in pancreatic cancer. In present study, we confirmed that FOXC2 knockdown remarkably inhibited PC cell proliferation and invasion, like that of CF129 overexpression. Furthermore, the re-overexpression of FOXC2 abrogated CF129-mediated inhibition on proliferation and migration of PC cells. Thus, these data suggest that FOXC2 promotes pancreatic cancer progression, which is a critical target for CF129. In agreement, the clinical samples verified a reversed correlation of FOXC2 and CF129, with short overall survival.

Many studies have found that lncRNAs can exert their effects in part via interactions with specific proteins. Therefore, we further applied bioinformation analysis to define the putative protein which might be involved in the regulation of CF129-FOXC2 pathway. The intersecting analysis of catRAPID and ChIPBase demonstrated that p53 protein might be recruited by both CF129 transcript and FOXC2 promoter. Coincidently, the RIP and RNA-pull down assays revealed that CF129 can bind to p53. Moreover, the ChIP and luciferase reporter assays verified the accumulation and activation of p53 on FOXC2 promoter, which was impeded by CF129 overexpression. In addition, p53 knockdown obviously reduced FOXC2 expression which was inhibited by CF129. Therefore, these results indicate that p53 is required for the regulation of CF129 on FOXC2. The accumulation of mutant p53 protein inactivates its anti-proliferative properties but promote tumorigenesis and invasion through a gain-of-function activity 23-25. The fact that TP53 is mutated and not deleted in PC and many other types of cancer indicates that this mutated form of p53 may provide some growth advantage to tumor cells. Coincidently, we also demonstrated that knockdown of p53 significantly suppressed proliferation and migration progression of BxPC-3 and PANC-1 cells, which possesses p53 mutation. Therefore, our data further supports that mutant p53 functions as promoter in pancreatic cancer.

The stability of p53 is carefully regulated via the ubiquitin-proteasome system, allowing levels of this protein to rise and fall in response to stressful conditions. lncRNAs are also known to play a role in regulating p53 26, with LncR-MALAT1 repressing p53 expression in a manner associated with cell cycle progression 27. In contrast, lncRNA-MEG3 promotes the activation of p53 in meningioma cell lines; a function associated with decreased MDM2 expression 28, 29. In addition, being a p53 effector, LncRNA- RoR can also repress p53 in response to DNA damage by interacting with hnRNP I, by which forming an autoregulatory feedback loop 30. Nevertheless, the precise mechanism about how these lncRNAs regulate the p53 expression needs further investigation. Differently, our present study demonstrated CF129 inhibited p53 expression in post-transcriptional level by enhancing its degradation.

Base on bioinformatic prediction from online database catRAPID, we revealed an association between CF129 and Makorin Ring Finger Protein 1 (MKRN1). MKRN1 is an E3 ligase for hTERT 31. Furthermore, it has recently been reported that MKRN1 induces the ubiquitination and degradation of p53 and p21 in a proteasome-dependent fashion 15. Therefore, we hypothesized that MKRN1 may play a role in CF129-meidated degradation of p53. Coincidently, the RIP and RNA-pull down assays verified an association of CF129 and MKRN1. Moreover, MKRN1 was validated to associate with p53 and induce its ubiquitination and degradation in PC cells. Furthermore, we found that the interaction between p53 and MKRN1 was enhanced by CF129 overexpression but reduced by the CF129 knockdown, which consequently impacted p53 ubiquitination and degradation. Nevertheless, the MKRN1 knockdown rescued the inhibition of CF129 on p53/FOXC2 expression, as well as the MKRN1 overexpression reversed the p53/FOXC2 overexpression which was upregulated by CF129 knockdown. These results raise the possibility that CF129 acts as bridge to mediates the MKRN1- dependent ubiquitination and degradation of p53. Similarly, lncRNA-uc.134 was reported to inhibit the CUL4A-mediated LATS1 ubiquitination, increasing YAPS127 phosphorylation leading to silencing of YAP gene targets 32. Therefore, our study affords further evidence for the lncRNA mediating ubiquitination of target protein.

Hypoxia is common in solid tumors including those in pancreatic cancer, and it can modulate tumor progression as well as therapeutic responses 33. Analyses from Hani et al. revealed that hypoxic conditions markedly affect RNA regulation, with hypoxia-inducible factor (HIF) serving as a primary regulator of this hypoxic transcriptome 34. Previous studies have found that many hypoxia-associated lncRNAs correlate with poor cancer prognoses. A nuclear lncRNA NEAT1 is regulated principally by HIF-2, which induces paraspeckle formation leads to accelerated cellular proliferation in hypoxia 35. Results displayed that HIF drives the expression of lncRNA-EFNA3 that causes Ephrin-A3 protein accumulation and contributes to metastatic spread of breast cancer 16. Coincidently, our previous results demonstrated that hypoxia-induced lncRNA-NUTF2P3-001 acts as competitive endogenous RNA (ceRNA) to depress the inhibition of miR-3923 on KRAS, which contributes to tumorigenesis of pancreatic cancer 9. On the contrary, histone deacetylase3 (HDAC3) repressed lncRNA-LET expression during hypoxia and stabilization of NF90 protein, which leads to hypoxia-induced cancer cell invasion 17. Similarly, our previous study discovered HDAC1 functions as co-factor with HIF-1α inhibits transcription of miR-548an 18. Since the present study displayed that CF129 is downregulated by hypoxia-induced HIF-1α, we further investigated whether HADAC1 is also involved in the hypoxia-induced downregulation of CF2129.Coincidently, our results demonstrate that HIF-1α transcriptionally downregulates CF129 expression by recruiting HDAC1 to the promoter of CF129 during hypoxia. Therefore, these results demonstrate a novel dysregulated lncRNA-CF129 of pancreatic cancer in response to hypoxia.

Given the HIF-1α promoter containing potential binding sites for FOXC2, we further assumed whether HIF-1α can be reciprocally regulated by FOXC2. In agreement, our data showed that siFOXC2 dramatically inhibited HIF-1α expression in both mRNA and protein level. Moreover, the recruitment of FOXC2 on HIF-1α HREs was further identified by ChIP and luciferase reporter assays. Furthermore, CF129 dramatically inhibited HIF-1α expression in hypoxic conditions, and FOXC2 overexpression reversed this. In contrast, CF129 knockdown significantly increased HIF-1α expression which is inhibited by siFOXC2 during hypoxia. This suggests that FOXC2 mediates a reciprocal regulation of CF129 and HIF-1α. A result from Fan et al. reported that hypoxia/HIF-1α-induced lincRNA-p21 can disrupt the VHL-HIF-1α interaction and reciprocally enhances HIF-1α accumulation, by which promoting glycolysis under hypoxia 36. Thus, our study supplies a novel data supporting a reciprocal regulation of lncRNA on HIF-1α transcription.

In summary, we have shown that CF129 inhibits PC cell proliferation and invasion by suppression of FOXC2 transcription, which depends on MKRN1- mediated ubiquitination and degradation of mutant p53. Our findings suggest that the CF129/MRKN1/ p53/FOXC2 axis may be a promising target for PC therapy.

## Methods

### Patients and tissue samples

In this study, 40 pancreatic cancer and corresponding non-tumor tissue samples were obtained from individuals undergoing operations at the Pancreatic Surgery Center, Union Hospital (Wuhan, China) from October 2015 to June 2016. If patients had previously undergone radiotherapy, chemotherapy or immunotherapy, they were not eligible for this study. All pancreatic cancer tissues were confirmed by the histopathological reports. Tumor staging was based on the revised international system. These clinical samples were used after obtaining informed consent from participants and their parents. All protocols were approved by the Union Hospital ethics committee.

### Cell culture

The PANC-1 and BxPC-3 cell lines came from American Type Culture Collection (ATCC, USA). These cell lines were cultivated in RPMI-1640 (HyClone) tinting with 10% FBS (ScienCell) as well as penicillin/streptomycin in a standard 37℃ incubator. To induce hypoxia, cells were cultured with 0.1% O_2_, 1% O_2_, 5% O_2_, 5% CO_2_ and corresponding 94.9% N_2_ , 94% N_2_, 90% N_2_ ,respectively or treated with CoCl_2_ (400 μmol/L). Total RNA and protein were collected after 0h, 6h, 12h, 24h and 48h.

### Transfection

The siRNA targeting CF129, FOXC2, p53, MKRN1, HIF-1α, HDAC1 and appropriate negative controls were obtained (Ribobio Co., Guangzhou, China). The plasmids containing CF129, FOXC2 and MKRN1 and the matched negative control plasmid were synthesized from GeneChem Co. (Shanghai, China). Transfections were performed with lipofectamine 2000 (Invitrogen) based on provided instructions. The siRNAs were transfected at a final concentration of 50nM. For plasmids, 1.6μg was transfected into samples in 12-well plate, while 0.2μg was used for 96 well plate. Protein and total RNA were extracted at 48 h post-transfection for downstream analyses.The siRNA sequences used in this study are all shown in **Supplementary Table 2.**

### qRT-PCR

An RNAiso Plus kit (TAKARA) was used for total RNA extraction based on provided protocols. The PrimeScript® RT Master Mix Perfect Real Time kit (TAKARA) was used to synthesize DNA. PCR amplifications were performed with the SYBR Premix Ex Taq II (TAKARA). The expression levels of LncRNA-CF129, FOXC2, p53, MKRN1, HIF-1α and HDAC1 were normalized to GAPDH as reference genes.Primer sequences used in this study are all shown in **Supplementary Table 3.**

### Cell proliferation assay

An MTT assay was employed to assess the proliferation of target cells. In 96-well plate, stable transfected cells (2000/well) with eight vices well for each sample. The proliferation was observed for 1 to 5 days. At certain hour every day,20μl MTT (5 mg/mL) was added to appropriate wells, and after 4h it was replaced with 150μl DMSO (Sigma). Finally, ELISA reader was used to determine the absorbance at 570nm.

### Assessment of migration and invasion

PANC-1 and BxPC-3 migratory abilities were measured using a wound healing assay. Stable transfected cells were allowed to form a confluent monolayer in 12-well plates. We observed the healing of the wound for 0h, 24h and 48h after would scratching with microscope. To invasion assay, the Matrigel Invasion Chamber of pore size 8μm (Corning, Shanghai, China) coated with Matrigel (BD Biosciences, Shanghai, China) was used. 5×10 cells in serum-free media were placed in the upper chamber with 250μL serum-free medium, while 700μL medium with 30% FBS was added to the lower chamber. After 24h, the cells were fixed and stained with removed in upper chamber. Finally, cells on the lower chamber were counted via microscopic assessment of five fields.

### Plate clone formation assay

Stable transfected cells in 6-well plates (1000/well) were cultured for 8 days. Wash cells with PBS and Fix cells with % polyformaldehyde for 20 minutes, and followed 1% Crystal Violet for 20 minutes after discarding solution. Finally, we count the number of cells in each well under the microscope.

### Fluorescence *in situ* hybridization

lncRNA CF129 was assessed via fluorescence in situ hybridization, a kit from named FISH Tag™ RNA Multicolor Kit (Invitrogen, USA) was used. The prepared cell samples were fixed with 5% formaldehyde for 25 minutes. Samples were then combined overnight at 42°C with the CF129 probe. Cells were then washed, nuclei were stained using DAPI, and stained cells were imaged via an LSM 5 Pascal Laser Scanning Microscope (Zeiss).

### RNA pull-down assay

The Pierce™ Magnetic RNA-Protein Pull-Down (Thermo Scientific, USA) and MAXIscript® (Ambion, USA) kits were used for this assay based on provided protocols. Briefly, PANC-1 nuclear extracts were combined with biotinylated CF129 or antisense- CF129, after which streptavidin beads were added to the samples. Pulled down RNA was then isolated by washing these beads, boiling them in SDS buffer, and subsequent western immunoblotting.

### RNA immunoprecipitation (RIP)

A Magna RIP™ RNA-Binding Protein Immunoprecipitation Kit (Millipore, USA) was used based on provided protocols. Briefly, 500uL lysis buffer supplemented with protease and RNase inhibitors was used to lyse cells in 6-well plates. Lysates were spin at 16,000 xg for 10 minutes, and supernatants were used for precipitation with antibodies against p53, MRKN1, and anti-mouse IgG or anti-rabbit IgG for 3 h at 4°C with gentle agitation. Beads were then washed three times in a wash buffer, twice in PBS, and RNA was isolated using the RNAiso Plus (TAKARA), for qRT-PCR as described above.

### Western Blotting and Co-immunoprecipitation (Co-IP)

Protein samples were electrophoretically separated on 10% polyacrylamide gels, followed by transfer to PVDF membranes (Millipore). Blots were then probed using the following primary antibodies: p53 (Proteintech,10442-1-AP), FOXC2 (Proteintech, 23066-1-AP), MKRN1 (Abcam, ab72054), HIF-1α (Proteintech, 20960-1-AP), HDAC-1 (Proteintech, 10197-1- AP), GAPDH (Proteintech, 10494-1-AP).

For co-IP analysis, anti-MKRN1, anti-p53 or normal mouse/rabbit IgG were used as the primary antibody, and then the antibody-protein complex was following incubated with Protein A/G PLUS-Agarose (Santa Cruz Biotechnology). The agarose-antibody- protein complex was collected and then analyzed by Western blot.

### Chromatin immunoprecipitation (ChIP)

The EZ-ChIPTM Chromatin Immunoprecipitation Kit (Millipore, Billerica, MA, USA) was used based on provided instructions. All the procedures were performed according to the manufacturer's instructions. Rabbit anti-HIF-1α/HDAC-1 was used and corresponding rabbit-IgG as controls. PCR was used to amplify bound DNA, which was then assessed via electrophoretic separation on a 2% agarose gel.

### Luciferase reporter assay

To investigate whether FOXC2 is transcriptionally regulated by p53, PANC-1 cells were transfected with FOXC2 promoter (P2/P3-WT or P2/P3-MUT) reporter constructs, and further co-transfected with sip53. After a 48-hour culture, the Dual-Luciferase reporter assay system was used to assess promoter activity, with Renilla luciferase used for normalization.

To determine how HIF-1α and HDAC1 affect the activity of HRE on the CF129 promoter under hypoxia condition. PANC-1 and BxPC-1 cells expressing pGL3-based construct containing the HRE of CF129 were transfected with siNC and siHIF-1α, and further treated with and without TSA. Twenty-four hours later, cells were cultured with or without CoCl2 for an additional 24h. The reporter activity was measured as above.

### Xenograft Mouse Model

PANC-1 cells that had been lentivirally transduced to express negative control (LV-NC) or CF129 sequences (LV-CF129) were injected into the dorsal flanks of 4-week-old male nude BALB/c mice. (HFK Bio-Technology Co., Ltd Beijing, China). (n=7 per group). Tumor growth in these animals was monitored every 3 days, and mice were euthanized 21 days after implant at which time tumor weights were determined.

In other experiments, mice instead received tail vein injections of stably transfected PANC-1 cells, and upon sacrifice the frequency and number of liver metastases in these mice was determined. The total RNA of tumors was extracted and analyzed by qRT-PCR. Studies on animals were conducted with approval from the Animal Research Committee of the Academic Medical Center of Huazhong University of Science and Technology.

### Statistical Analysis

SPSS v13.0 and GraphPad Prism were utilized for all data analyses. Data are given as Mean ± SD. Comparisons between 2 groups were made with Student's t-tests, while survival was assessed via Kaplan-Meier approach with a Log-rank test. The Wilcoxon signed-rank test was used to assess significant differences of CF129, p53 and FOXC2 between samples. χ2 tests were utilized to analyze the relationships between lncRNA-CF129 levels and clinical characteristics. P<0.05 was the threshold of significance.

## Supplementary Material

Supplementary figures and tables.
