# Supplementary material for: Hypoxia-induced feedback of HIF-1α and lncRNA-CF129 contributes to pancreatic cancer progression through stabilization of p53 protein
Source: Theranostics. 2019 Jul 9;9(16):4795–810. doi: 10.7150/thno.30988 (PMC6643431; doi:10.7150/thno.30988)
Supplement: Supplementary file 1 — Supplementary figures and tables. [file thnov09p4795s1.pdf]

Supplementary Figure Legends

Supplementary Figure 1 CF129 depletion promotes PC cell proliferation and invasion. (A) Three different CF129-siRNAs and corresponding negative control (NC-siRNAs) were designed and the significantly effective two siRNAs (siCF129-1 and siCF129-2) were chosen to fulfill the following experiments. (B, C) The effects of siCF129 on proliferation of PANC-1/BxPC-3 cells was determined with MTT and colony formation assays. (D, E) The effect of knockdown of CF129 on PC cell migration and invasion was investigated by wound-healing and transwell assays.

Supplementary Figure 2 FOXC2 is necessary for PC cell proliferation and invasion. (A) After transfected with siFOXC2-1 or siFOXC2-2, the protein expression of FOXC2 in PANC-1/BxPC-3 cells was measured by western blotting. (B) The effect of siFOXC2 on PANC-1/BxPC-3 cell proliferation was investigated via MTT assay. (C) siFOXC2 effects on PANC-1/BxPC-3 cell invasion were measured via transwell assay. (D) After transfected with siCF129 or/and siFOXC2, FOXC2 protein levels in BxPC-3 cells were detected via western blotting. (E) The proliferation of BxPC-3 cells after siCF129 or/and FOXC2 transfection were compared by MTT assay. (F) The transwell assay displayed the invasion ability of BxPC-3 cells after siCF129 or/and siFOXC2 transfection.

Supplementary Figure 3 CF129 regulates FOXC2 transcription through association with p53. (A, B) FOXC2 levels were detected in siCF129 or/and sip53 transfected BxPC-3 cells via qRT-PCR and western blotting. (C) The proliferation of BxPC-3 cells following siCF129 or/and sip53 transfection were compared via MTT assay. (D) siCF129 or/and sip53 transfected BxPC-3 cell invasion was detected via transwell assay.

Supplementary Figure 4 CF129 depletion repairs ubiquitination and degradation of p53. (A, B) After transfected with siCF129, PANC-1/BxPC-3 p53 levels were assessed via western blotting and qRT-PCR. (C) After siCF129 transfection, PANC-1/BxPC-3 cells were treated using CHX (100 μg/mL) and then p53 levels were assessed via western blotting. Band intensities that reflected the remaining p53 protein levels were quantified by software (ImageJ and GraphPad) (D) siCF129-transfected PANC-

1/BxPC-3 cells were cultured with MG132(20nM) for 3h, subjected to western blot. (E) After transfected with siCF129, PANC-1/BxPC-3 cell lysates underwent anti-p53 immunoprecipitation and immunoblotting with anti-ubiquitination antibody.

Supplementary Figure 5 The MKRN1 is required for the CF129 -mediated p53 degradation. (A) After 3 hour MG132 treatment, PANC-1 cells underwent immunoprecipitation using anti-p53 or -MKRN1, and were then immunoblotted using anti-MKRN1 (Left) or -p53 antibody (Right). (B) The expression of p53 and MKRN1 protein in siMKRN1-transfected PANC-1/BxPC-3 cells were measured by western blot. (C) Western blot analysis of p53 expression in PANC-1/BxPC-3 cells after siCF129 or/and MKRN1-OE transfection. (D) After transfection using siCF129, the lysates of PANC-1/BxPC-3 cells underwent immunoprecipitation as in (A). (E) After transfected with CF129-si or/and MKRN1-OE, the lysates from PANC-1/BxPC-3 cells underwent immunoprecipitation with anti-p53 antibody and immunoblotting analysis with anti-ubiquitination antibody. (F) After transfected with siMKRN1 and sip53, the association between CF129 with MKRN1 or p53 were detected by RIP and western blot.

Supplementary Figure 6 CF129 is downregulated by HIF-1α during Hypoxia. (A) CoCl2 was used for treating BxPC-3 cells, and expression of CF129 and HIF-1α were detected by qRT-PCR and western blotting, respectively, at the indicate timepoints. BxPC-3 cells were transfected using siNC and siHIF-1α during treatment with normoxia or CoCl2. (B) CF129 expression in those BxPC-3 cells was detected by qRT-PCR. (C) Single molecule RNA FISH displayed the CF129 expression (green) in those BxPC-3 cells. Nucleus was counterstained with DAPI. (D) ChIP assays were performed with those transfected BxPC-3 cells using anti-HIF-1α antibody. (E) Those BxPC-3 cells were co-transfected using WT or MUT plasmids as reporter cells. Luciferase density of these cells was detected and normalized. (F) The expression of HIF-1α, p53, and FOXC2 proteins in those BxPC-3 cells was assessed via western blot. (G) After co-treated with CF129-OE and CoCl2, the expression of p53 and FOXC2 in BxPC-3 cells was evaluated by western blot.

Supplementary Figure 7 Effects of CF129 on pancreatic cancer cells proliferation under different oxygen concentration (A) The effects of CF129 on

proliferation of PANC-1 cells was evaluated by MTT assay during 5%O_2. (B) The effects of CF129 on proliferation of PANC-1 cells was evaluated by MTT assay during 1%O_2 . (C) The effects of CF129 on proliferation of PANC-1 cells were determined by MTT assay during 0.1%O_2).

Supplementary Figure 8 Histone deacetylase 1 (HDAC1) is involved in the HIF-1α-induced reduction of CF129 expression during hypoxia. (A) The effects of siHDAC1 on HDAC1 levels were evaluated in PANC-1/BxPC-3 cells by qRT-PCR and western blotting. (B) CF129 expression was measured in PANC-1/BxPC-3 cells following transfection with either siNC or siHDAC1 during normoxia or hypoxia conditions. (C) The expression of CF129 was measured in PANC-1/BxPC-3 cells, which were treated using TSA or control DMSO during normoxia or hypoxia conditions. (D) ChIP assays were performed in BxPC-3 cells following siNC or siHDAC1 transfection by using anti-HDAC1 antibody under normoxia or hypoxia condition. (E) ChIP assays were performed in TSA- or DMSO-treated BxPC-3 cells by using anti-HDAC1 antibody under normoxia or hypoxia condition. (F) BxPC-3 cells were transfected using WT or MUT plasmid as reporter cells, followed by siNC or siHDAC1 transfection under normoxic or hypoxic conditions. The luciferase activity was measured and normalized. (G) The reporter BxPC-3 cells were treated with DMSO or TSA during normoxia or hypoxia condition. The luciferase activity was detected and normalized.(H) The protein levels of HDAC1, p53,and FOXC2 were measured in HDAC1-knockdown BxPC-3 cells during normoxia or hypoxia condition.(I) The protein levels of HDAC1, p53, and FOXC2 were measured in TSA- or DMSO-treated BxPC-3 cells during normoxia or hypoxia condition.

Supplementary Figure 9 The correlation between CF129 and FOXC2 in pancreatic cancer samples. (A) FOXC2 mRNA levels in PC tissue samples (n=40) and paired non-cancerous pancreatic tissue samples. (B, C) FOXC2 expression levels in PC tissue samples (n=36) and non-cancerous pancreatic tissues (n=16) from GSE16515 data. (D) Scatter diagram exhibited the correlation between CF129 and FOXC2 in 40 paired pancreatic cancer tissues via qRT-PCR. (F) The correlation between FOXC2 and HIF-1α mRNA was assessed in pancreatic cancer tissues from TCGA database using a Pearson’s correlation analysis. (G) Kaplan-Meier analysis of

the association between FOXC2 level and overall survival of PC patients. * $P < 0.05$,**$P < 0.01$.

Supplementary Figure 1

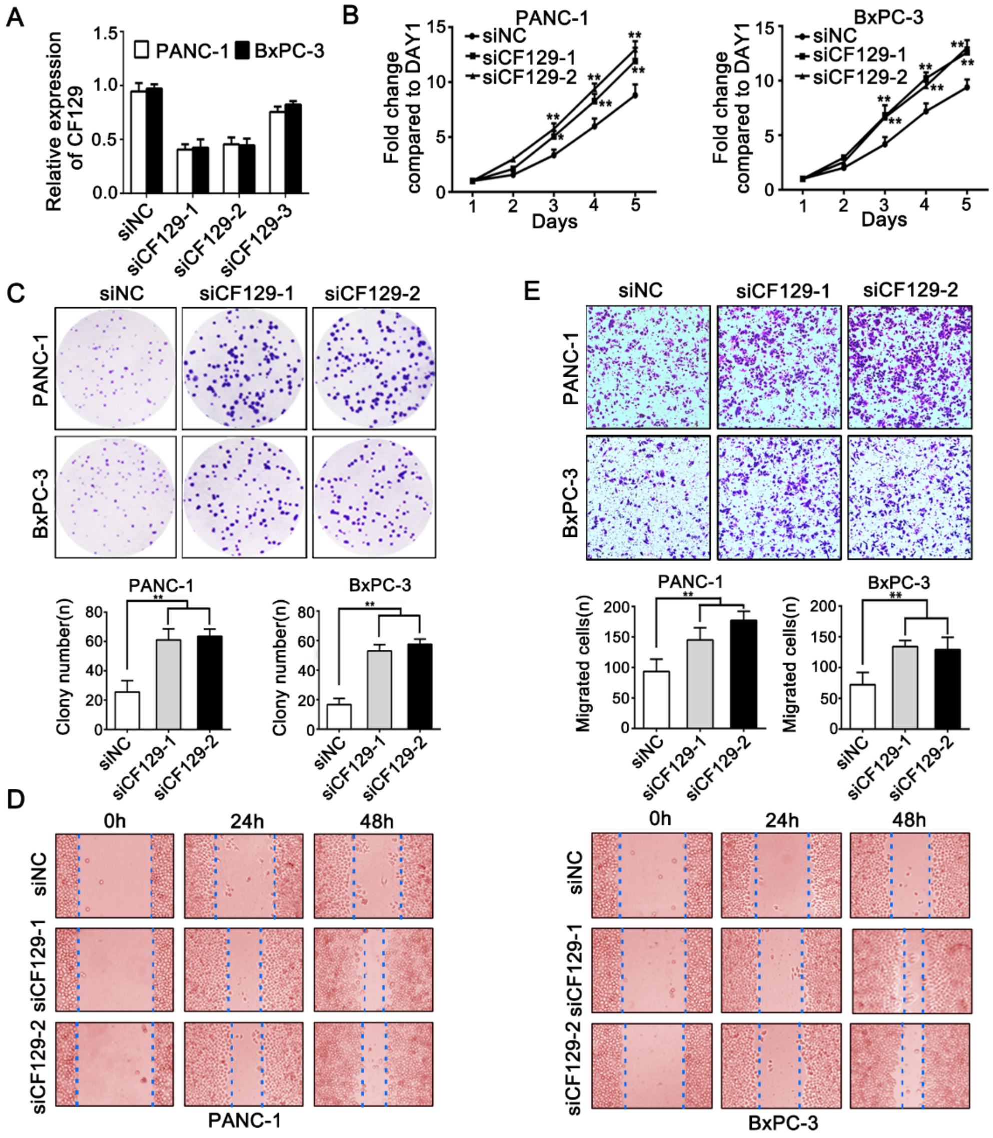

Supplementary Figure 2

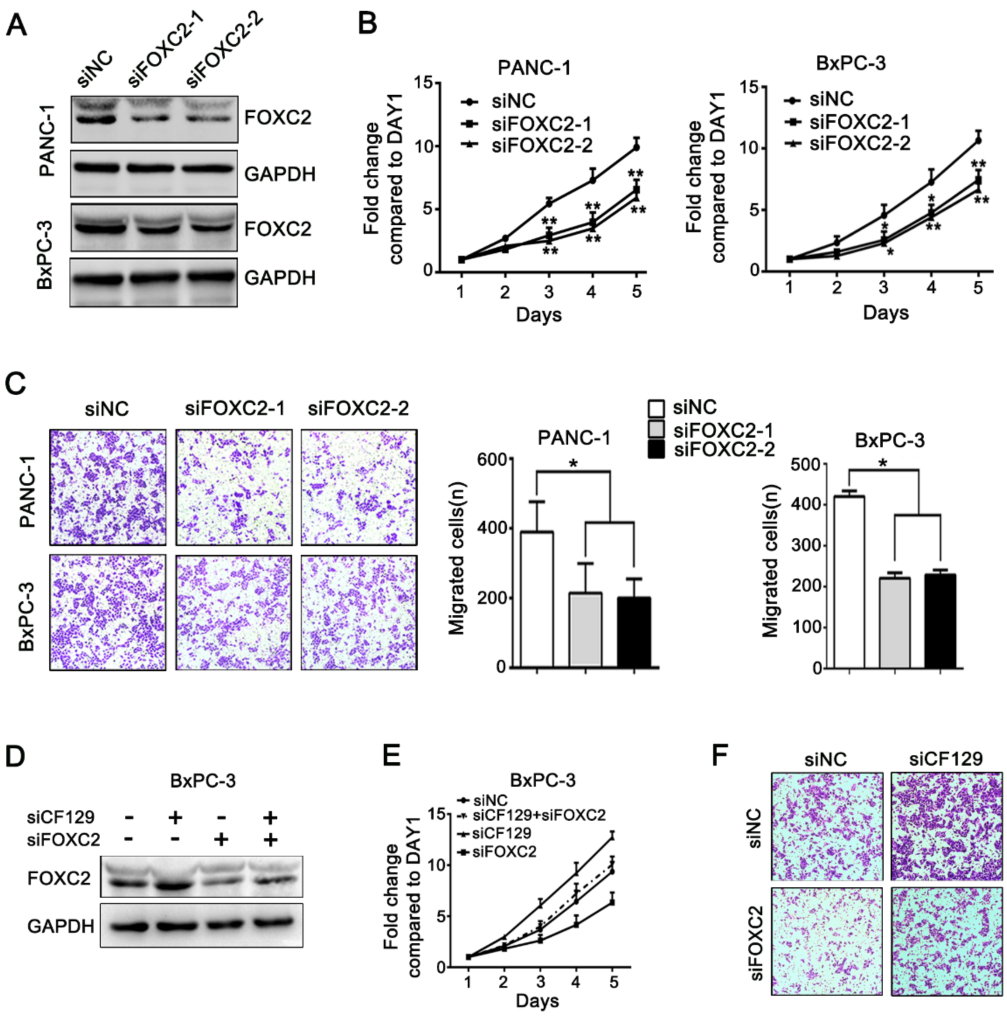

Supplementary Figure 3

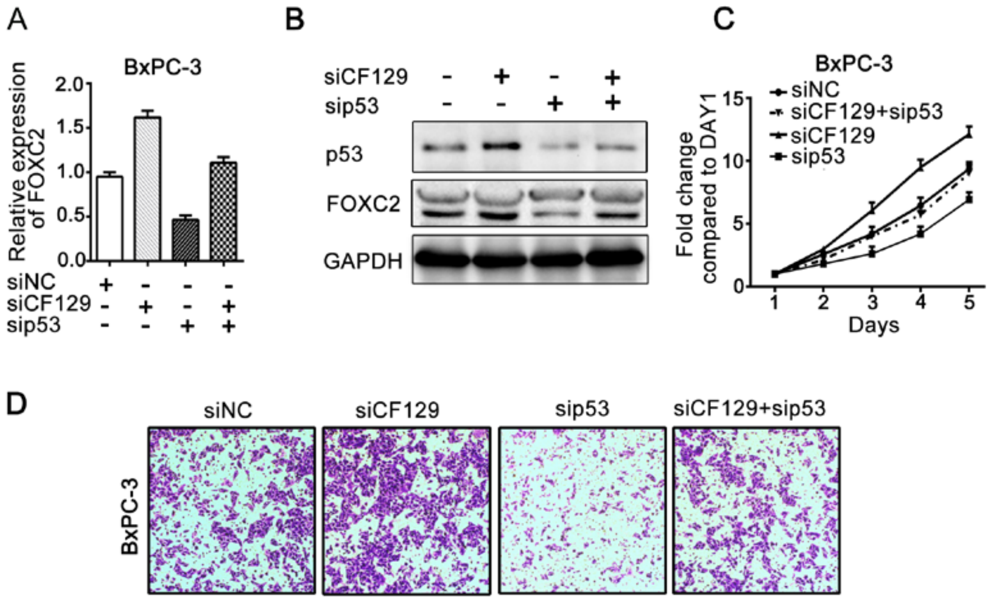

Supplementary Figure 4

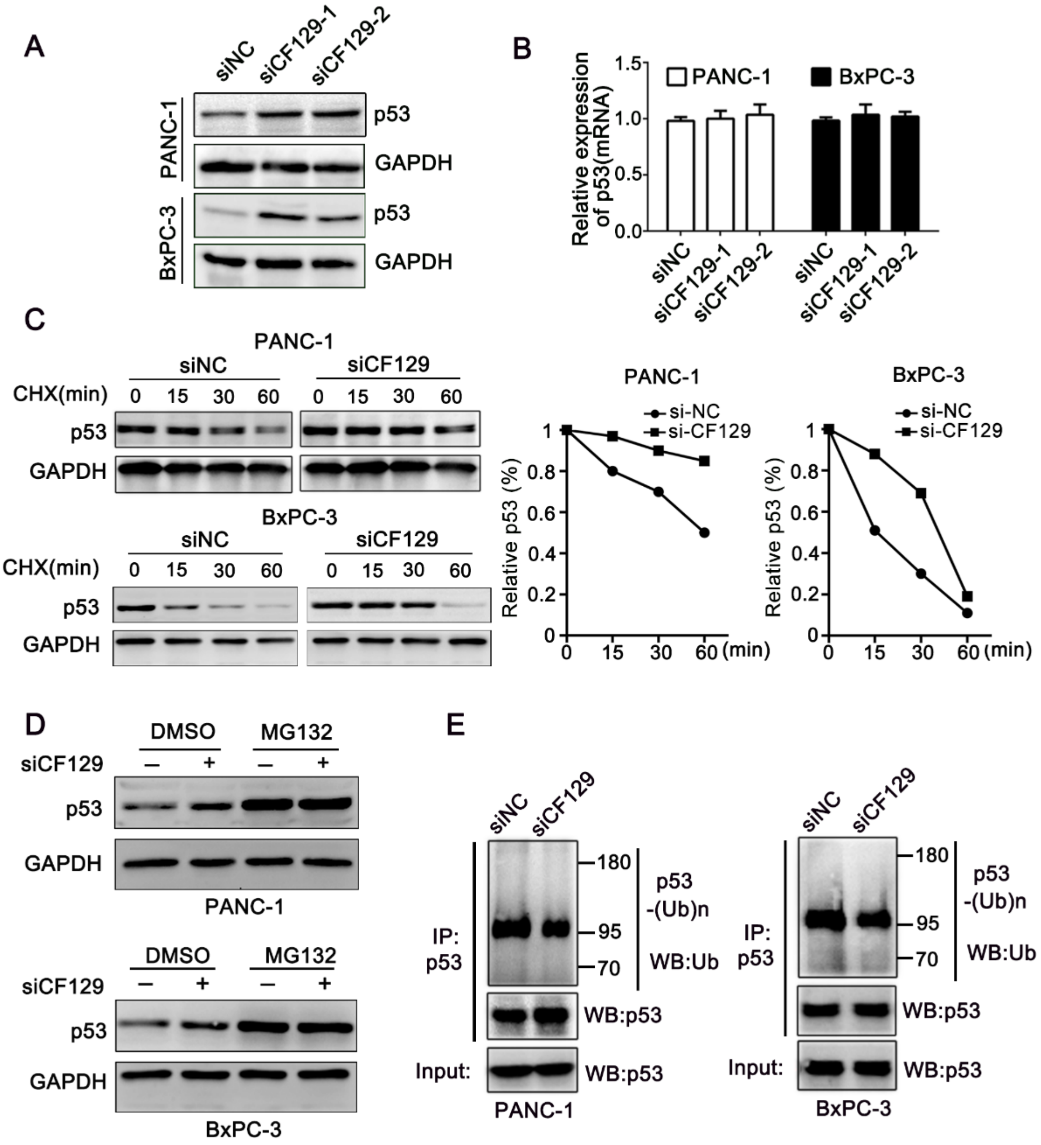

Supplementary Figure 5

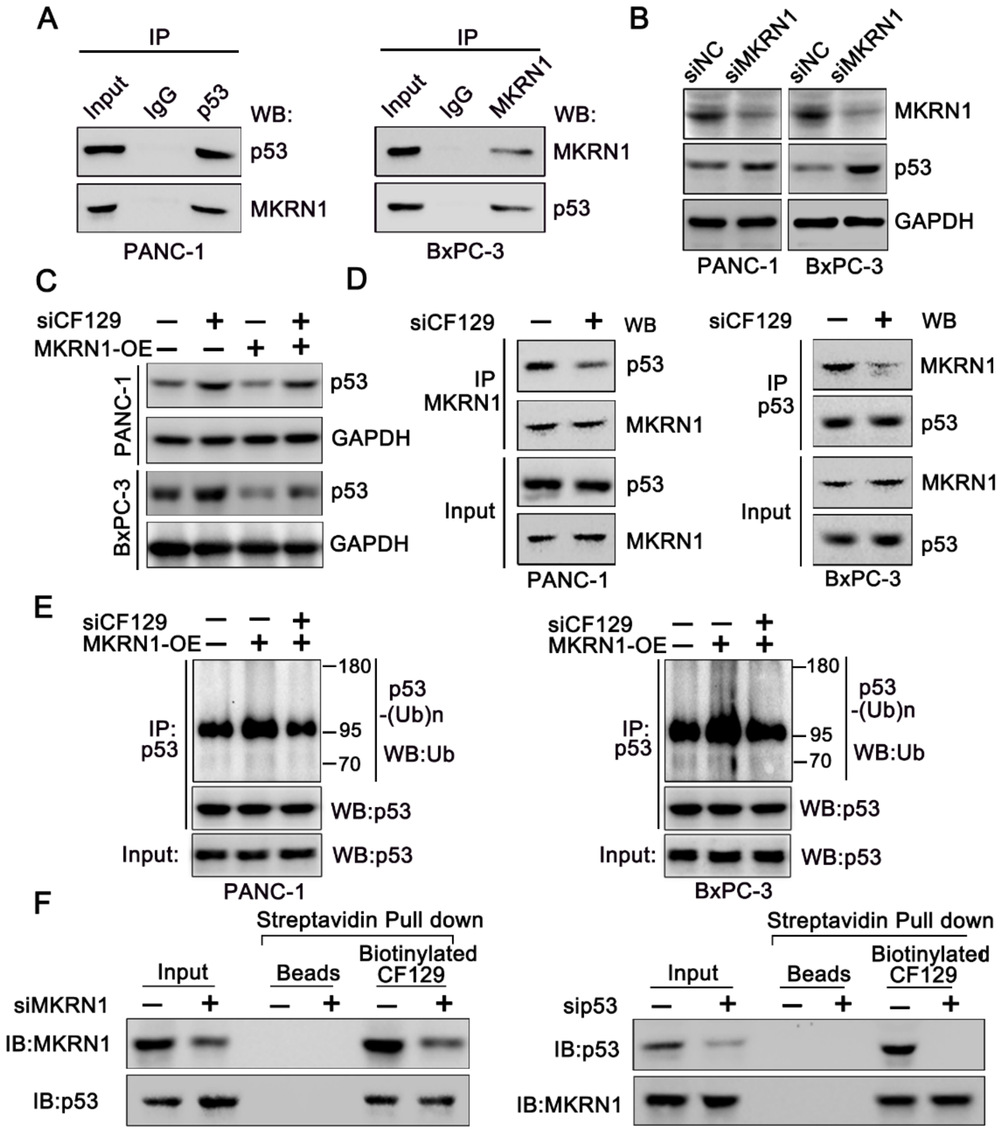

Supplementary Figure 6

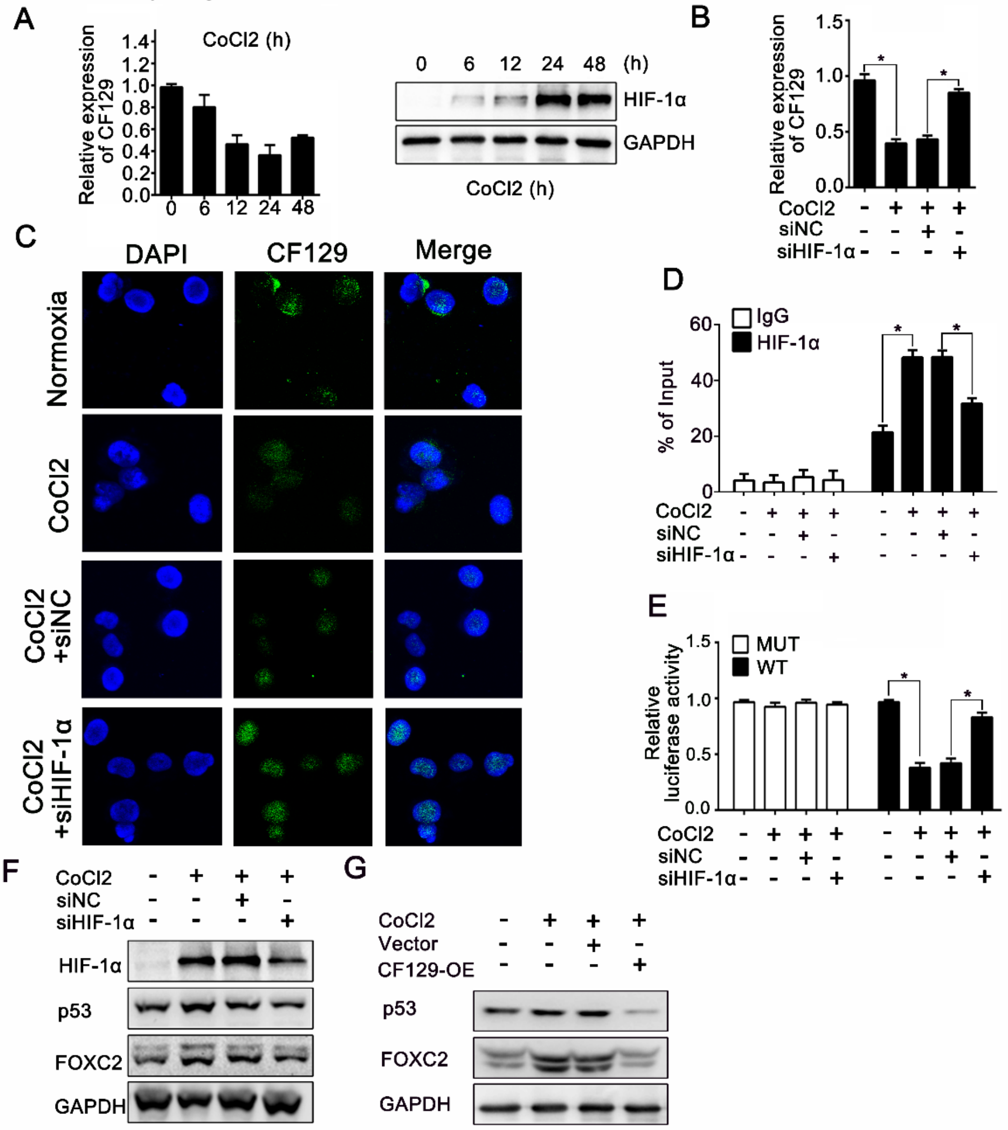

Supplementary Figure 7

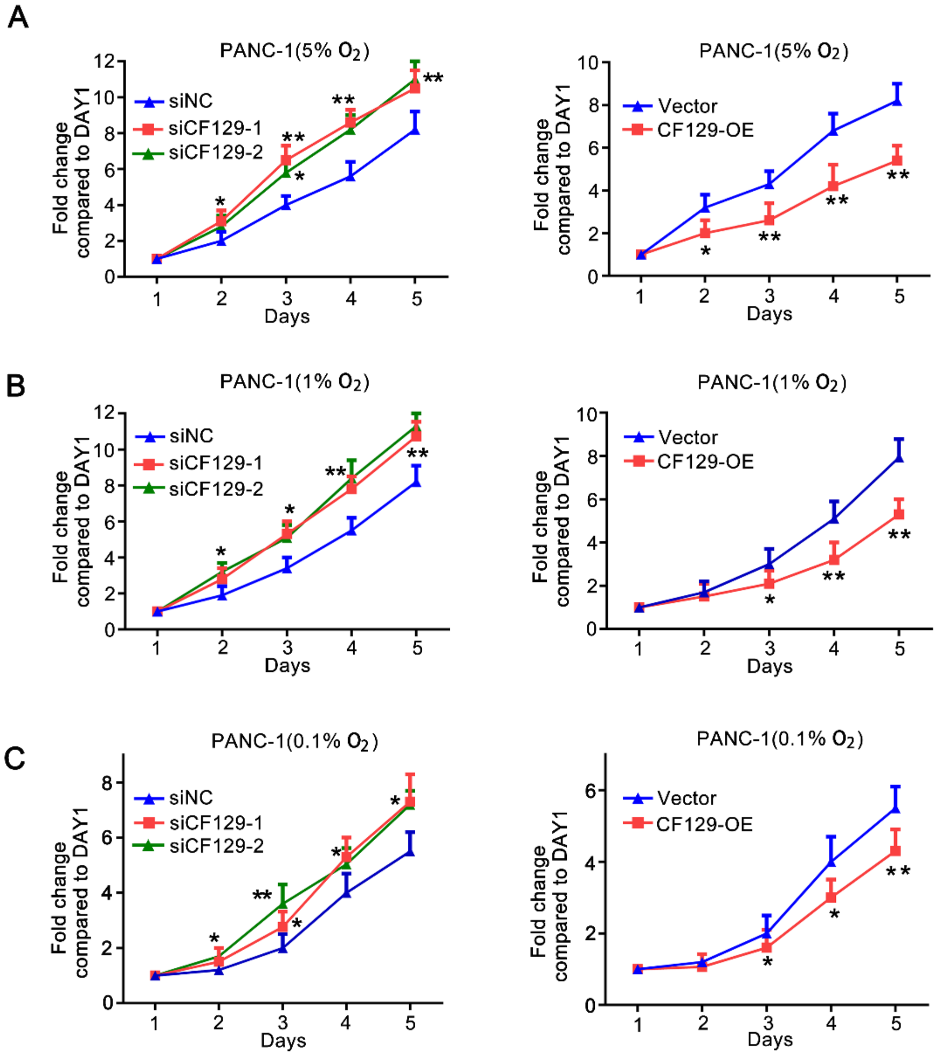

Supplementary Figure 8

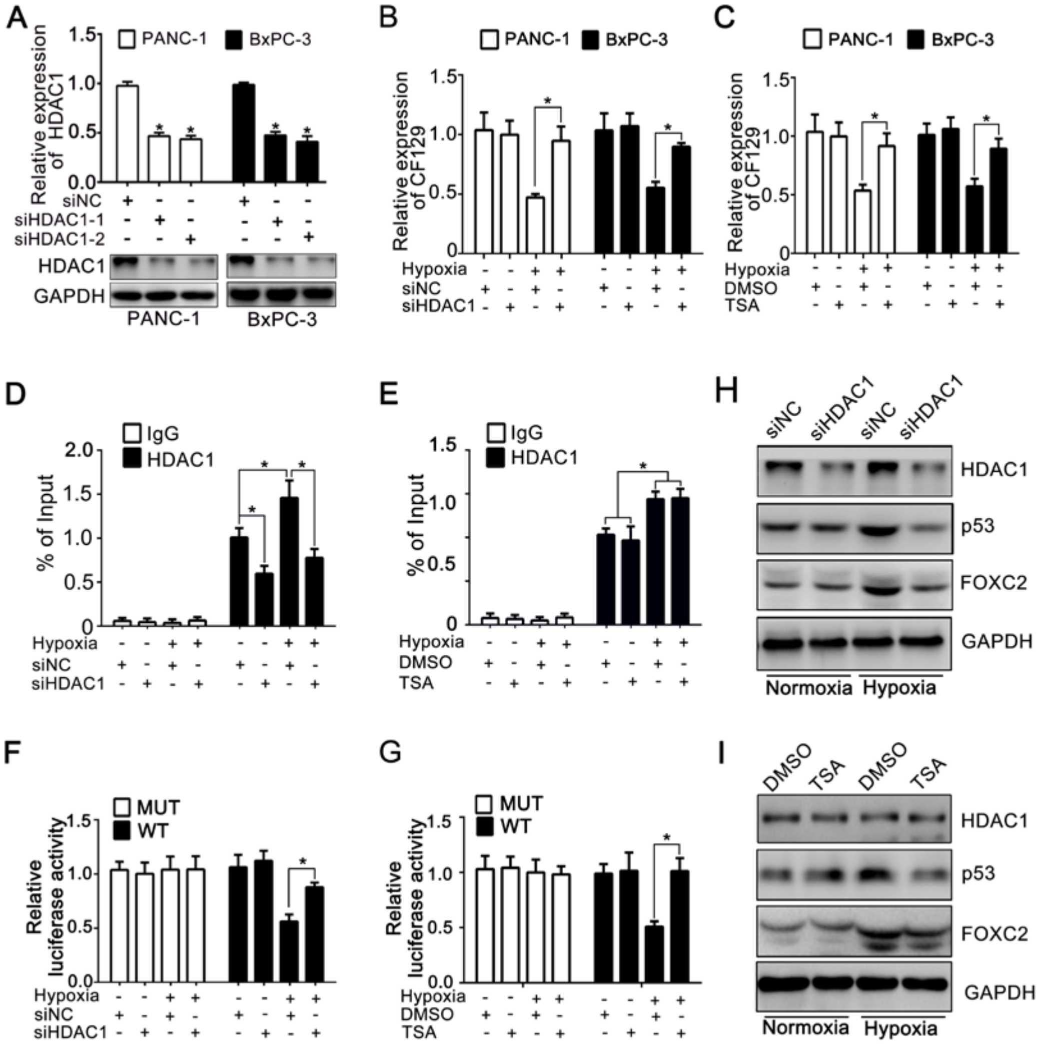

Supplementary Figure 9

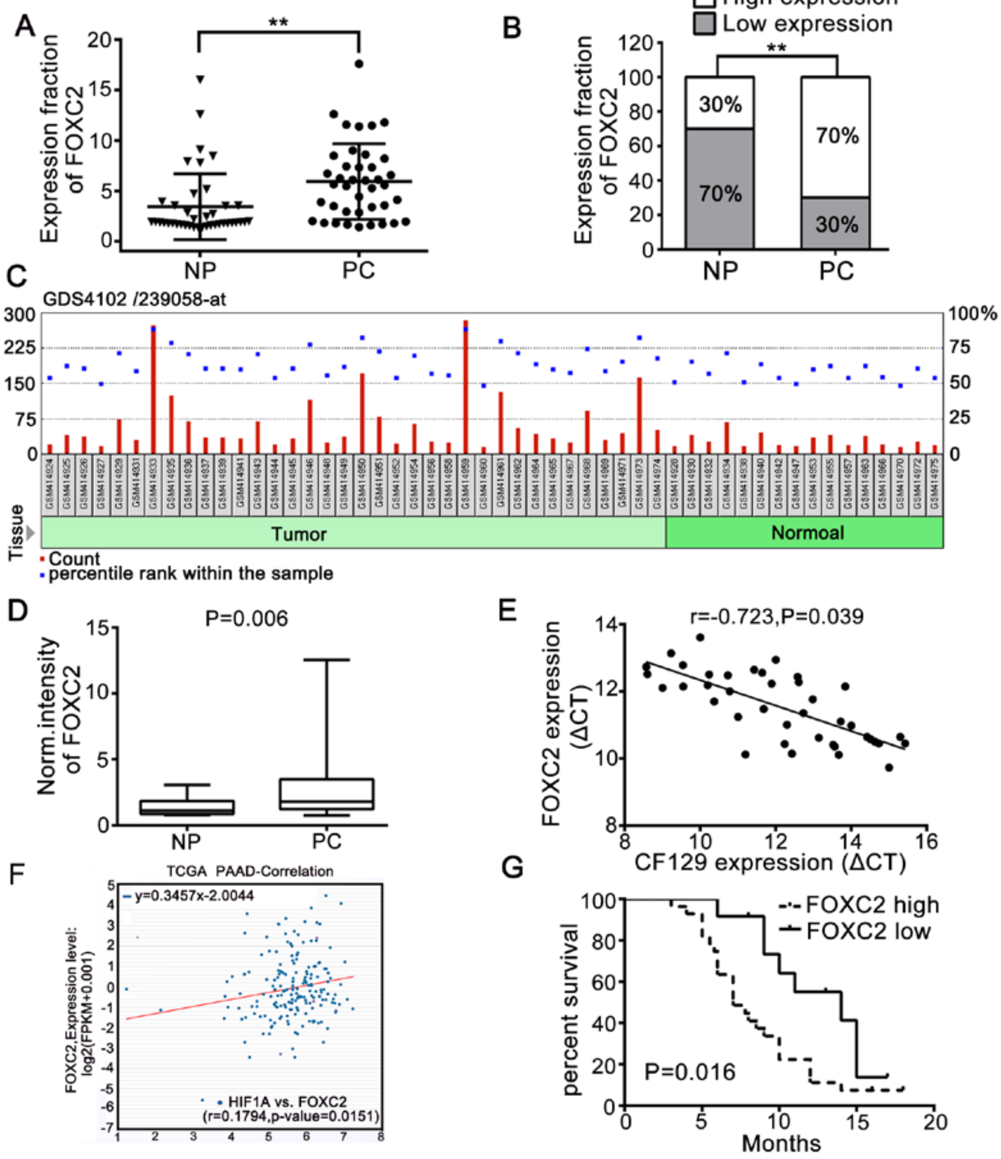

Supplementary Table 1 Clinical characteristics of 40 PC patients according to LncRNA CF129 expression levels

Feature	LncRNA CF129 expression		χ2	P value*
	Low	High		
**All cases**	20	20		
**Age**			0.902	0.342
<60	8	11		
≥60	12	9		
**Gender**			1.616	0.204
Male	9	13		
Female	11	7		
**Tumor size (cm)**			5.013	0.025
<2	5	12		
≥2	15	8		
**Histological Grade**			6.465	0.011
High/Moderate	7	15		
Low	13	5		
**TNM Stage**			0.96	0.327
I–II	11	14		
III–IV	9	6		
**Lymphatic invasion**				
Positive	12	4	6.667	0.01
Negative	8	16		
**Vascular infiltration**				
Positive	14	11	0.96	0.327
Negative	6	9		
**Distant metastasis**			7.033	0.008
Positive	11	3		
Negative	9	17		

# The median expression level was used as the cutoff. Low CF129 expression in each of the 40 patients was defined as a value below the 50th percentile. High CF129 expression in each of the 40 patients was defined as a value above the 50th percentile.

* For analysis of correlation between the expression of CF129 and clinical features, Pearson chi-square tests were used. Results were considered statistically significant at P < 0 .05.

Supplementary Table 2: The sequences of siRNA

Targets	SS Sequence	AS Sequence
[illegible]-1	GGUUAAUGUUAACAUCACAAA	UGUGAUGUUAACAUUAACCUG
[illegible]-2	CUGUGUUAGUAUAGUGAAACU	UUUCACUAUACUAACACAGAA
[illegible]-3	GUGUGUAUGUUAAAGAAUUAG	AAUUCUUUAACAUACACACGG
[illegible]2-1	CCUACAACAUGUUCGAGAACG	UUCUCGAACAUGUUGUAGGAG
[illegible]2-2	AGAAGAAGGUGGUGAUCAAGA	UUGAUCACCACCUUCUUCUCG
[illegible]	GGUGUUACUUCCUGAUAAACU	UUUAUCAGGAAGUAACACCAU
[illegible]1	GCUGAGUCAAGAAAUUCAAAC	UUGAAUUUCUUGACUCAGCUU
[illegible]	CGAUGGAAGCACUAGACAAAG	UUGUCUAGUGCUUCCAUCGGA
[illegible]1-1	GGUGGAGGUUGCUAGUCUAGU	UAGACUAGCAACCUCCACCUG
[illegible]1-2	CAGCGAUGACUACAUUAAAUU	UUUAAUGUAGUCAUCGCUGUG

Supplementary Table 3: Primers for qRT-PCR and ChIP assays

Targets	Sequences
CF129 forward	5′-TTGTGCCCGTTTGAATGGTG-3′
CF129 reverse	5′-GCAGCTGGTTACTGGAAACG-3′
FOXC2 forward	5′-TTCATCATGGACCGCTTCCC-3′
FOXC2 reverse	5′-CGCTCTTGATCACCACCTTCT-3′
p53 forward	5’-CCAGGGAGCACTAAGCGAGCA-3’
p53 reverse	5’-GTCTGAGTCAGGCCCTTCTGT-3’
MKRN1 forward	5’-GAGAAGGACATGGAGCTCTCA-3’
MKRN1 reverse	5’-CGCCTTGTTGCTCATTGCCTC-3’
HIF-1α forward	5′-CAAGATCTCGGCGAAGCAA-3′
HIF-1α reverse	5′-GGTGAGCCTCATAACAGAAGCTTT-3′
HDAC1 forward	5′-TAAATTCTTGCGCTCCATCC-3′
HDAC1 reverse	5′-AACAGGCCATCGAATACTGG-3′
GAPDH forward	5′-GAAGGTGAAGGTCGGAGTC-3′
GAPDH reverse	5′-GAAGATGGTGATGGGATT-3′
FOXC2 P1 forward	5′-AATACGCAGCCGATGAAC-3′
FOXC2 P1 reverse	5′-CAGACCTGGAGCGACTTG-3′
FOXC2 P2/P3 forward	5′-AATCAACCGCTCACCCAG-3′
FOXC2 P2/P3 reverse	5′-GACACTCGCAGCCTACCAA-3′
CF129 HRE forward	5′-GAAAGCACGATGAGTTAA-3′
CF129 HRE reverse	5′-GCTATTCGGATTGGTAAG-3′
